# Supplementary material for: A core genome MLST scheme for Borrelia burgdorferi sensu lato improves insights into the evolutionary history of the species complex
Source: Cell Rep Methods. 2024 Dec 18;5(1):100935. doi: 10.1016/j.crmeth.2024.100935 (PMC11840949; doi:10.1016/j.crmeth.2024.100935)
Supplement: Document S1. Figures S1 and S2 and Tables S4–S7 [file mmc1.pdf]

Cell Reports Methods, Volume *5*

Supplemental information

A core genome MLST scheme for *Borrelia burgdorferi* sensu lato improves insights into the evolutionary history of the species complex

Sabrina Hepner, Keith A. Jolley, Santiago Castillo-Ramirez, Evangelos Mourkas, Alexandra Dangel, Andreas Wieser, Johannes Hübner, Andreas Sing, Volker Fingerle, and Gabriele Margos

Supplementary information

Supplemental figures

Figure S1: Example of the assignment of the core genome sequence type (cgST) and clustering to core genome clusters (Bb_cgc), related to STAR Methods.

Figure S2: Minimum spanning tree based on the cgMLST scheme using 294 isolates, related to Table 3 and Figure 4C-D.

Supplemental tables

Table S4: Distance matrix of the Asian *B. bavariensis* isolates (n=30) based on MLST, related to Table 2 and Figure 4.

Table S5: Distance matrix of the Asian *B. bavariensis* isolates (n=30) based on cgMLST, related to Table 2 and Figure 4.

Table S6: Distance matrix of the European *B. bavariensis* isolates (n=19) based on MLST, related to Table 2 and Figure 4.

Table S7: Distance matrix of the European *B. bavariensis* isolates (n=19) based on cgMLST, related to Table 2 and Figure 4.

Supplemental figures

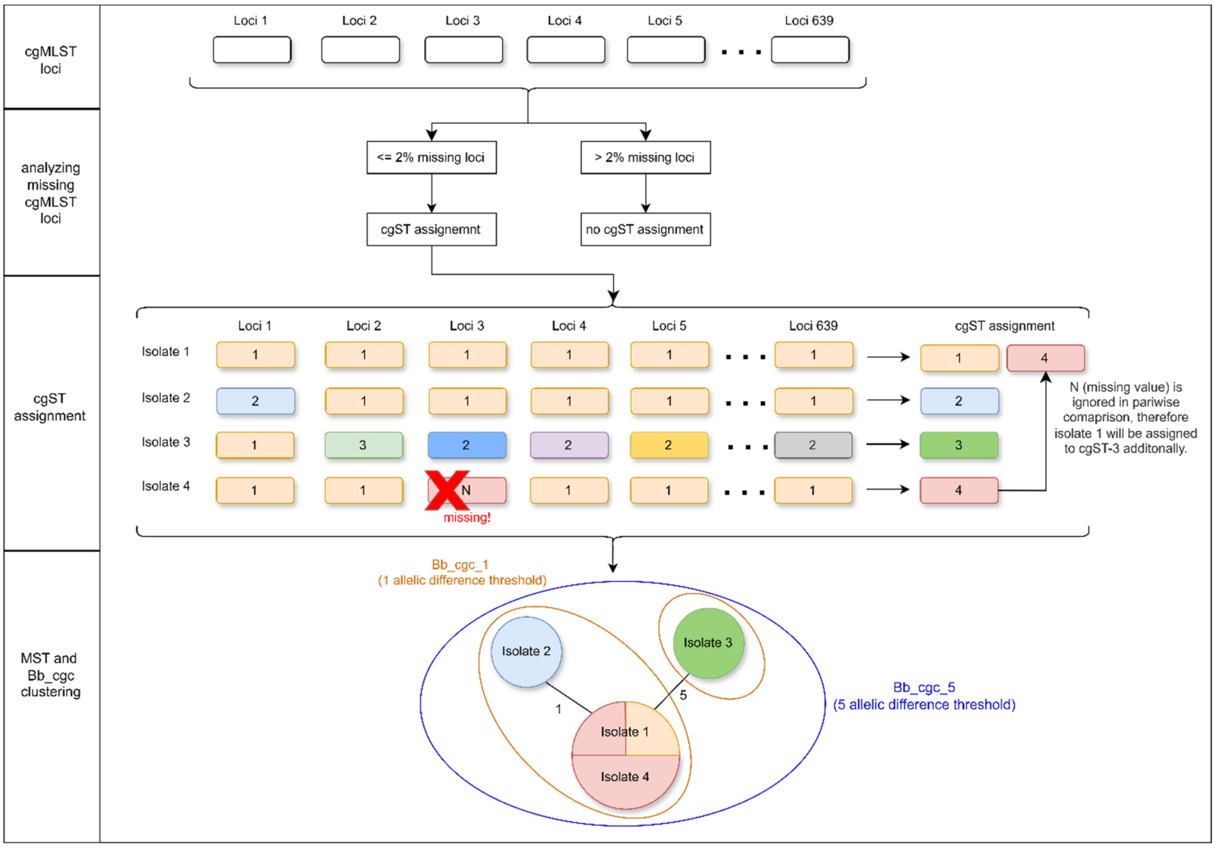

Figure S1: Example of the assignment of the core genome sequence type (cgST) and clustering to core genome clusters (Bb_cgc), related to STAR Methods.

In isolate 1, 2, 3 and 4 <= 2% of the cgMLST loci were missing resulting in cgST assignment. Isolate 1 with a new unique allelic profile (no missing values) is assigned to cgST-1. Isolate 2 differs in loci 1 and therefore has a new unique profile and is assigned to cgST-2. Isolate 3 has a new unique allelic profile and is assigned to cgST-3. Isolate 4 has the identical allele profile as isolate 1, but loci 3 is missing and therefore will be assigned with “N”. As it is not known which allele is missing, a new cgST-4 will be assigned. As missing values (“N”) will be ignored in pairwise comparison, isolate 1 will also match cgST-4 and also receive this designation. Comparing the isolates regarding allelic differences, isolate 1 and isolate 4 has 0 allelic differences, while these two isolates has one allelic difference to isolate 2 and five allelic differences to isolate 3 which can be visualized in the MST. Applying a threshold of five allelic differences, all isolates belong the same Bb_cgc_5 cluster, while they fell in two different Bb_cgc_1 clusters using a threshold of one allelic difference.

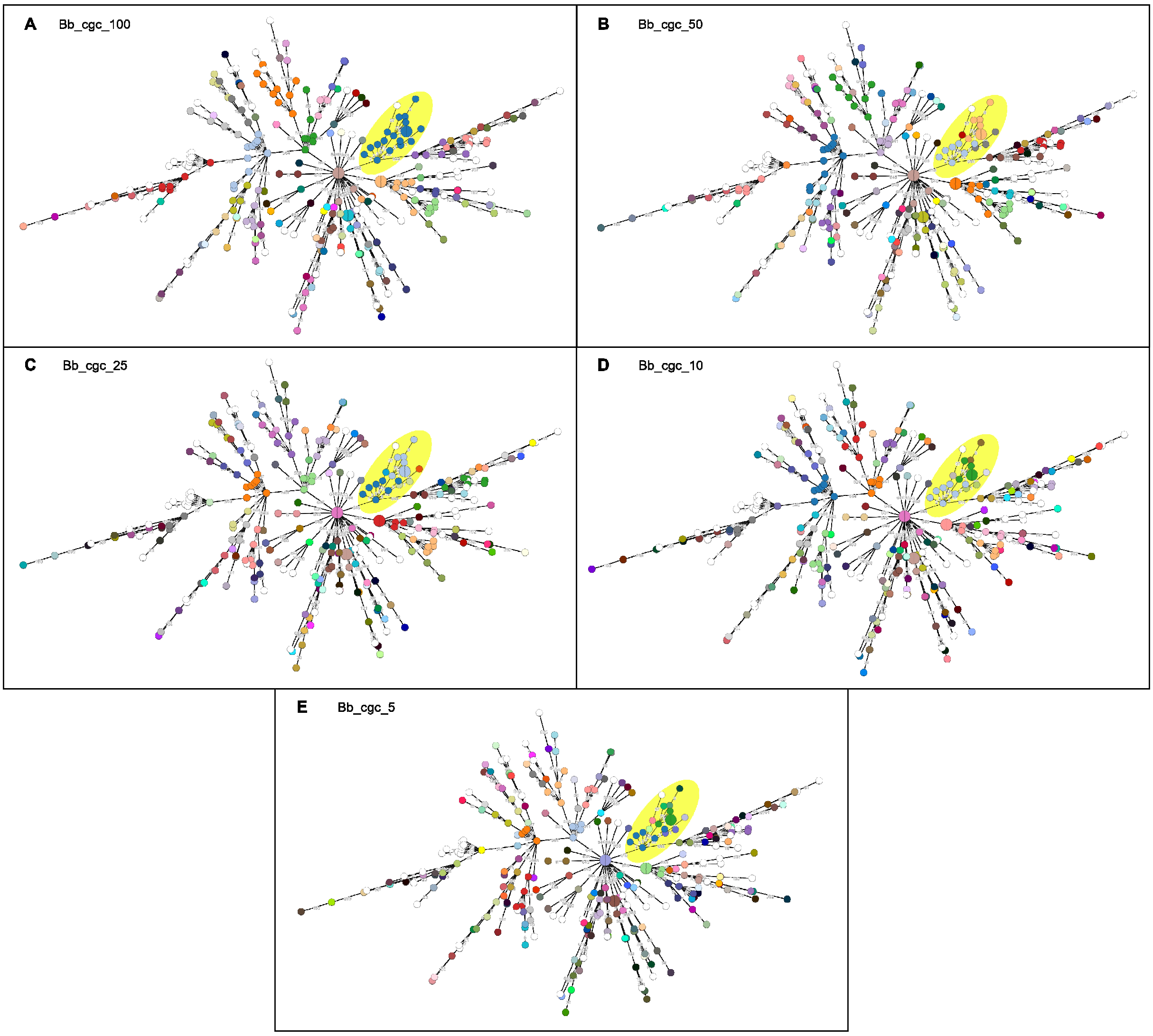

Figure S2: Minimum spanning tree based on the cgMLST scheme using 294 isolates, related to Table 3 and Figure 4C-D.

MST are colored according to *B. burgdorferi* core genome cluster (Bb_cgc) applying various allelic differences thresholds: (A) Bb_cgc_100, (B) Bb_cgc_50, (C) Bb_cgc_25, (D) Bb_cgc_10, (E) and Bb_cgc_5. Isolates with missing cgST are shown in white.

Supplemental tables

Table S4: Distance matrix of the Asian *B. bavariensis* isolates (n=30) based on MLST, related to Table 2 and Figure 4.

Arh913-2012_wgs																														
Arh923-2012_wgs	8																													
FujiP2_wgs	3	7																												
Hiratsuka_wgs	8	0	7																											
J-14_wgs	3	7	0	7																										
J-15_wgs	8	8	8	8	8																									
J-20T_wgs	8	8	8	8	8	8																								
konnai_17_clone1_wgs	1	8	3	8	3	8	8																							
N346_wgs	8	8	8	8	8	8	8	8																						
Prm965-2013_wgs	8	7	8	7	8	8	8	8	8																					
Prm7019-12_wgs	8	8	8	8	8	8	8	8	8	7																				
Prm7564-11_wgs	8	1	8	1	8	8	8	8	8	7	8																			
Prm7569-11_wgs	8	7	8	7	8	8	8	8	8	0	7	7																		
BgVir_wgs	8	8	8	8	8	8	8	8	8	8	8	8	8																	
NMJW1_wgs	1	8	3	8	3	8	8	0	8	8	8	8	8	8																
SZ_wgs	1	8	3	8	3	8	8	0	8	8	8	8	8	8	0															
JAASAAF1014_wgs	8	8	8	8	8	8	8	8	8	7	7	8	7	8	8	8														
JAASAAF1016_wgs	8	0	7	0	7	8	8	8	8	7	8	1	7	8	8	8	8													
JAASAAF1029_wgs	1	8	3	8	3	8	8	0	8	8	8	8	8	8	0	0	8	8												
JAASAAM1091_wgs	8	8	8	8	8	8	8	8	8	7	7	8	7	8	8	8	0	8	8											
JAASAAM1101_wgs	3	7	0	7	0	8	8	3	8	8	8	8	8	8	3	3	8	7	3	8										
JAASAAM1114_wgs	8	0	7	0	7	8	8	8	8	7	8	1	7	8	8	8	8	0	8	8	7									
JANAGAM1271_wgs	3	7	0	7	0	8	8	3	8	8	8	8	8	8	3	3	8	7	3	8	0	7								
JANAGAM1274_wgs	3	7	0	7	0	8	8	3	8	8	8	8	8	8	3	3	8	7	3	8	0	7	0							
JANAGAM1305_wgs	3	7	0	7	0	8	8	3	8	8	8	8	8	8	3	3	8	7	3	8	0	7	0	0						
JANAGAM1327_wgs	3	7	0	7	0	8	8	3	8	8	8	8	8	8	3	3	8	7	3	8	0	7	0	0	0					
JANAGAM1352_wgs	8	0	7	0	7	8	8	8	8	7	8	1	7	8	8	8	8	0	8	8	7	0	7	7	7	7				
JANAGAM1353_wgs	8	0	7	0	7	8	8	8	8	7	8	1	7	8	8	8	8	0	8	8	7	0	7	7	7	7	0			
JANAGAM1369_wgs	3	7	0	7	0	8	8	3	8	8	8	8	8	8	3	3	8	7	3	8	0	7	0	0	0	0	7	7		
NT24_wgs	3	7	0	7	0	8	8	3	8	8	8	8	8	8	3	3	8	7	3	8	0	7	0	0	0	0	7	7	0	

Table S5: Distance matrix of the Asian *B. bavariensis* isolates (n=30) based on cgMLST, related to Table 2 and Figure 4.

Arh913-2012_wgs																														
Arh923-2012_wgs	624																													
FujiP2_wgs	159	624																												
Hiratsuka_wgs	626	177	626																											
J-14_wgs	160	624	12	626																										
J-15_wgs	630	635	631	635	631																									
J-20T_wgs	632	634	631	634	631	632																								
konnai_17_clone1_wgs	98	623	155	625	155	630	631																							
N346_wgs	631	630	633	630	633	631	633	631																						
Prm965-2013_wgs	626	623	626	624	626	633	631	626	633																					
Prm7019-12_wgs	629	626	628	628	628	633	632	628	632	555																				
Prm7564-11_wgs	624	25	624	180	624	635	634	623	630	623	626																			
Prm7569-11_wgs	625	624	625	626	625	633	630	625	632	61	558	624																		
BgVir_wgs	608	623	610	625	609	632	632	607	628	625	625	623	624																	
NMJW1_wgs	98	623	149	625	149	630	631	43	631	626	628	623	625	607																
SZ_wgs	96	623	147	625	147	630	631	40	631	626	628	623	625	607	3															
JAASAAF1014_wgs	626	630	627	631	627	630	634	626	629	630	633	630	631	630	626	626														
JAASAAF1016_wgs	625	181	625	70	625	635	634	624	630	624	627	183	625	625	624	624	628													
JAASAAF1029_wgs	94	624	150	626	150	630	631	23	631	627	628	624	626	607	42	39	626	625												
JAASAAM1091_wgs	626	630	627	631	627	629	634	626	628	629	632	630	630	630	626	626	16	628	626											
JAASAAM1101_wgs	168	624	25	626	25	631	631	163	633	626	628	624	625	610	157	155	627	625	159	627										
JAASAAM1114_wgs	624	194	624	119	624	635	634	623	629	624	627	196	625	624	623	623	628	109	624	628	624									
JANAGAM1271_wgs	177	625	35	627	35	631	631	172	633	627	628	625	626	609	165	165	627	626	166	627	46	624								
JANAGAM1274_wgs	242	625	122	627	123	632	631	235	633	627	629	625	626	613	232	231	628	625	235	628	131	625	137							
JANAGAM1305_wgs	166	624	19	626	19	631	631	160	633	626	628	624	625	609	154	152	627	624	156	627	30	623	41	129						
JANAGAM1327_wgs	176	624	34	626	27	631	631	171	633	626	628	624	625	609	167	165	626	624	166	626	46	621	53	133	41					
JANAGAM1352_wgs	619	330	621	268	620	634	635	618	631	627	629	334	627	626	618	618	624	235	620	624	619	290	619	620	619	613				
JANAGAM1353_wgs	624	199	624	129	624	635	634	623	629	623	627	200	625	624	623	623	626	119	624	626	624	65	621	624	623	622	296			
JANAGAM1369_wgs	165	624	18	626	18	631	631	159	633	626	628	624	625	609	153	151	627	624	154	627	29	623	40	129	7	38	619	623		
NT24_wgs	166	624	20	626	20	631	631	159	633	626	629	624	625	609	154	152	627	624	156	627	31	623	42	130	6	42	619	623	8	

Table S6: Distance matrix of the European *B. bavariensis* isolates (n=19) based on MLST, related to Table 2 and Figure 4.

PBi																			
A91S_wgs	0																		
DK6_wgs	0	0																	
Lubl25_wgs	1	1	1																
PNeb_wgs	0	0	0	1															
PNi_wgs	0	0	0	1	0														
PRab_wgs	0	0	0	1	0	0													
PRof_wgs	0	0	0	1	0	0	0												
PTrob_wgs	1	1	1	0	1	1	1	1											
PWin_wgs	0	0	0	1	0	0	0	0	1										
PZwi_wgs	1	1	1	0	1	1	1	1	0	1									
61VB2_wgs	0	0	0	1	0	0	0	0	1	0	1								
PBae_I_wgs	0	0	0	1	0	0	0	0	1	0	1	0							
A104S_wgs	0	0	0	1	0	0	0	0	1	0	1	0	0						
PBae_II_wgs	0	0	0	1	0	0	0	0	1	0	1	0	0	0					
PBar_wgs	0	0	0	1	0	0	0	0	1	0	1	0	0	0	0				
PBN_wgs	0	0	0	1	0	0	0	0	1	0	1	0	0	0	0	0			
PHer_I_wgs	0	0	0	1	0	0	0	0	1	0	1	0	0	0	0	0	0		
PLad_wgs	0	0	0	1	0	0	0	0	1	0	1	0	0	0	0	0	0	0	

Table S7: Distance matrix of the European *B. bavariensis* isolates (n=19) based on cgMLST, related to Table 2 and Figure 4.

PBi																			
A91S_wgs	107																		
DK6_wgs	12	104																	
Lubl25_wgs	83	90	82																
PNeb_wgs	74	83	72	57															
PNi_wgs	3	110	15	86	77														
PRab_wgs	77	87	75	62	9	80													
PRof_wgs	2	109	14	85	76	1	79												
PTrob_wgs	83	92	82	35	55	85	59	84											
PWin_wgs	72	83	71	58	4	75	7	74	55										
PZwi_wgs	82	91	81	34	54	84	58	83	1	54									
61VB2_wgs	11	106	10	81	72	14	75	14	81	71	80								
PBae_I_wgs	73	82	71	56	3	76	8	75	53	3	52	71							
A104S_wgs	176	114	173	165	154	179	153	178	165	154	164	176	154						
PBae_II_wgs	74	83	72	58	4	75	9	74	55	4	54	72	3	155					
PBar_wgs	84	94	82	70	18	87	20	86	68	18	67	82	17	157	18				
PBN_wgs	3	110	15	86	77	0	80	1	85	75	84	14	76	179	75	87			
PHer_I_wgs	74	84	72	58	5	77	10	76	55	5	54	72	2	156	5	19	77		
PLad_wgs	13	118	23	94	85	15	85	14	92	83	91	23	84	178	85	94	15	85	
