# Supplementary material for: A core genome MLST scheme for Borrelia burgdorferi sensu lato improves insights into the evolutionary history of the species complex
Source: Cell Rep Methods. 2024 Dec 18;5(1):100935. doi: 10.1016/j.crmeth.2024.100935 (PMC11840949; doi:10.1016/j.crmeth.2024.100935)
Supplement: Document S2. Article plus supplemental information [file mmc5.pdf]

Cell Reports
Methods

Article

A core genome MLST scheme for *Borrelia burgdorferi* sensu lato improves insights into the evolutionary history of the species complex

Graphical abstract

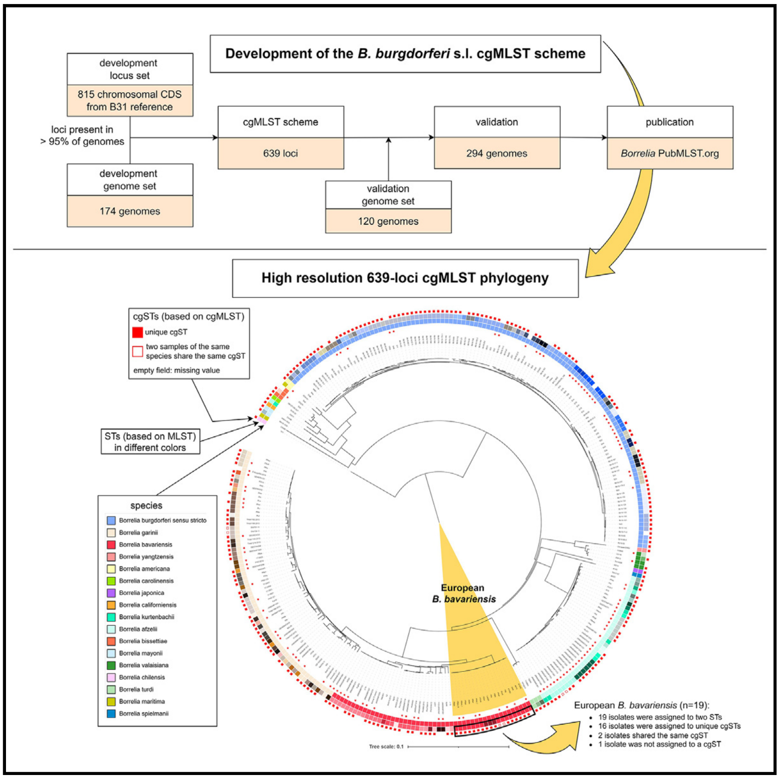

Highlights

- A core genome multi-locus sequence typing scheme for *Borrelia burgdorferi* sensu lato
- The scheme allows unambiguous genotyping and enables robust, high-resolution phylogenies
- We demonstrate high discriminatory power within species and closely related isolates
- The cgMLST scheme is publicly available on the *Borrelia* PubMLST website

Authors

Sabrina Hepner, Keith A. Jolley, Santiago Castillo-Ramirez, ..., Andreas Sing, Volker Fingerle, Gabriele Margos

Correspondence

sabrina.hepner@lgl.bayern.de

In brief

Hepner et al. develop a 639-loci core genome multi-locus sequence typing (cgMLST) scheme for *Borrelia burgdorferi* sensu lato that extends the traditional 8-loci MLST to a genomic scale. The cgMLST scheme gains higher reliability, robustness, and resolution, enabling improved insights into the evolutionary history of the species complex.

Check for updates

Hepner et al., 2025, Cell Reports Methods *5*, 100935
January 27, 2025 © 2024 The Authors. Published by Elsevier Inc.
https://doi.org/10.1016/j.crmeth.2024.100935

CellPress

Cell Reports Methods

CellPress
OPEN ACCESS

Article

A core genome MLST scheme for *Borrelia burgdorferi* sensu lato improves insights into the evolutionary history of the species complex

Sabrina Hepner,[1,2,11,*] Keith A. Jolley,[3] Santiago Castillo-Ramirez,[4] Evangelos Mourkas,[5] Alexandra Dangel,[2] Andreas Wieser,[6,7,8,9] Johannes Hübner,[10] Andreas Sing,[1,2] Volker Fingerle,[1,2] and Gabriele Margos[1,2]
[1]German National Reference Centre for Borrelia, Oberschleissheim, Germany
[2]Bavarian Health and Food Safety Authority, Oberschleissheim, Germany
[3]Department of Biology, University of Oxford, Oxford, UK
[4]Programa de Genómica Evolutiva, Centro de Ciencias Genómicas, Universidad Nacional Autónoma de México, Mexico City, Mexico
[5]Zoonosis Science Centre, Department of Medical Sciences, Uppsala University, Uppsala, Sweden
[6]Medical Microbiology and Hospital Epidemiology, Max von Pettenkofer Institute, Faculty of Medicine, LMU Munich, Munich, Germany
[7]Division of Infectious Diseases and Tropical Medicine, LMU University Hospital, LMU Munich, Munich, Germany
[8]German Center for Infection Research (DZIF), Partner Site Munich, Munich, Germany
[9]Immunology, Infectious Disease and Pandemic Research (IIP), Fraunhofer Institute for Translational Medicine and Pharmacology (ITMP), Munich, Germany
[10]Dr. von Hauner Children's Hospital, LMU Munich, Munich, Germany
[11]Lead contact
*Correspondence: sabrina.hepner@lgl.bayern.de
https://doi.org/10.1016/j.crmeth.2024.100935

MOTIVATION Multi-locus sequence typing (MLST) analyses based on eight chromosomal housekeeping genes (*clpA*, *clpX*, *nifS*, *pepX*, *pyrG*, *recG*, *rplB*, *uvrA*) are extensively used for *Borrelia* typing, population genetics, and evolutionary analyses. However, we still encounter limitations with regard to statistical support of inner nodes in phylogenies and the resolution of lineages within species. As whole-genome sequencing has become available, MLST can be extended to the genomic scale, analyzing a higher number of loci and grouping them in an extended scheme. We aimed to gain higher reliability, robustness, and resolution and have developed a 639-loci core genome MLST (cgMLST) scheme for *B. burgdorferi* sensu lato (s.l.) that is applicable to whole-genome data. The schemes are available on the *Borrelia* PubMLST database (https://pubmlst.org/organisms/borrelia-spp).

SUMMARY

Multi-locus sequence typing (MLST) based on eight genes has become the method of choice for *Borrelia* typing and is extensively used for population studies. Whole-genome sequencing enables studies to scale up to genomic levels but necessitates extended schemes. We have developed a 639-loci core genome MLST (cgMLST) scheme for *Borrelia burgdorferi* sensu lato (s.l.) that enables unambiguous genotyping and improves the robustness of phylogenies and lineage resolution within species. Notably, all inner nodes of the cgMLST phylogenies had consistently high statistical support. Analyses of the genetically homogeneous European *B. bavariensis* population support the notion that cgMLST provides high discriminatory power even for closely related isolates. While isolates differed maximally in one MLST locus, there were up to 179 cgMLST loci differences. Thus, the developed cgMLST scheme for *B. burgdorferi* s.l. resolves lineages at a finer resolution than MLST and improves insights into the evolutionary history of the species complex.

INTRODUCTION

Lyme borreliosis is the most prevalent tick-borne disease in the Northern Hemisphere, including North America and the temperate regions of Eurasia. The causative agents of the disease are spirochetes of the *Borrelia burgdorferi* sensu lato (s.l.) species complex.[1–3] The complex contains 22 species, including the six human pathogenic species that can cause Lyme borreliosis: *Borrelia afzelii*, *B. bavariensis*, *B. burgdorferi* sensu stricto (s.s.), *B. garinii*, *B. mayonii*, and *B. spielmanii*.[1,4–6] The bacterium

© 2024 The Authors. Published by Elsevier Inc.
This is an open access article under the CC BY license (http://creativecommons.org/licenses/by/4.0/).

Table 1. Overview of number of isolates per genospecies that was used to develop and validate the cgMLST scheme

Species	No. of isolates in the development genome set	No. of isolates in the validation genome set	No. of isolates in total
B. afzelii	6	31	37
B. americana	1	1	2
B. bavariensis	27	22	49
B. bissettiae	2	1	3
B. burgdorferi s.s.	86	34	120
B. californiensis	1	–	1
B. carolinensis	1	–	1
B. chilensis	1	–	1
B. garinii	37	29	66
B. japonica	1	–	1
B. kurtenbachii	1	–	1
B. maritima	1	–	1
B. mayonii	2	–	2
B. spielmanii	1	–	1
B. turdi	3	–	3
B. valaisiana	2	2	4
B. yangtzensis	1	–	1
Total	174	120	294

See also Table S1.

is maintained in transmission cycles between tick vectors of the *Ixodes ricinus-persulcatus* species complex and vertebrate reservoir hosts.[1,6,7]

The genome of *B. burgdorferi* s.l. is relatively small (~1.5 Mb) but remarkable for bacteria in that it consists of a linear chromosome and numerous linear and circular plasmids.[8–10] Sequence typing has become the state-of-the-art method for bacterial characterization and for the analyses of ecological and evolutionary processes that influence population structure and dynamics. Chromosomal as well as plasmid-located loci have been used for *B. burgdorferi* s.l. typing, but early analyses were mostly based on single-locus analyses (16S rRNA gene [*rrs*], *flaB*, *ospA*, *ospC*, *rrs-rrlA* intergenic spacer [IGS]).[11,12]

In 1998, a novel and portable bacterial typing technique was introduced: multi-locus sequence typing (MLST).[13] This approach used nucleotide sequences of multiple core genes, and it was suspected that MLST can be applied to almost all bacterial species. An advantage of MLST is that the method can be based on PCR amplification of the appropriate loci; thus, in the case of vector-borne bacteria, it can be applied to environmental samples (vector, host, or patient) and does not necessarily rely on cultured material.[12] Today, this approach has become the method of choice for many organisms and is extensively used in studies of population biology and public health surveillance of bacterial pathogens.[14–17] The sequence data are shared worldwide via MLST databases such as the PubMLST database (https://pubmlst.org/) or the Institute Pasteur MLST databases (https://bigsdb.pasteur.fr/).[17,18]

The *Borrelia* MLST scheme is based on eight chromosomal housekeeping genes (*clpA*, *clpX*, *nifS*, *pepX*, *pyrG*, *recG*, *rplB*, *uvrA*).[12,19] The major advantage of analyzing multiple loci in comparison to single loci is that a skewed evolutionary picture can be mitigated. Additionally, it allows the detection of, and can buffer against, recombination. Furthermore, the use of multiple loci leads to a higher resolution of lineages within species and improved epidemiological delineations.[12,15] Since 2015, the PubMLST.org website has been the home for *Borrelia* MLST/MLSA data[20] and currently contains over 78,000 unique allele sequence definitions and information for more than 3,900 isolates (https://pubmlst.org/organisms/borrelia-spp, June 26, 2024).

Even though MLST was a significant improvement over single-locus analyses, we still encounter some limitations—mainly due to limited statistical support of inner nodes in phylogenies, which may become more pronounced if some loci fail to produce typing results.[15,21,22]

Rapid and high-throughput sequencing methods by next-generation sequencing (NGS) technologies have opened the door for accurate whole-genome data, opening up new possibilities in terms of typing methods.[15] To take full advantage of this, new platforms for data storage and bioinformatics analyses as well as extended typing schemes are required. The Bacterial Isolate Genome Sequence Database (BIGSdb) is incorporated in the PubMLST.org website and allows the hosting of all levels of sequence data (single sequences up to whole-genome data) and comparative genome analyses.[17,18,20] This allows extending the principle of MLST to a higher number of loci that can be grouped into an extended typing scheme, known as core genome MLST (cgMLST).[15,18] It has been shown in other bacteria that cgMLST can lead to insights in the emergence and evolution of pathogens and enables standardized molecular surveillance, including a replicable typing method (for example, *Neisseria*,[23,24] *Listeria monocytogenes*,[25] *Mycobacterium tuberculosis*,[26] *Staphylococcus*,[27,28] *Campylobacter*,[29] *Salmonella*,[30–32] and *Klebsiella pneumonia*[33,34]). To extend the advantages of MLST to a genomic scale for *Borrelia*, we developed a 639-loci cgMLST scheme for *B. burgdorferi* s.l. that enables unambiguous genotyping and reliable phylogenetic analysis with high resolution. The scheme is publicly accessible through the *Borrelia* PubMLST.org website.

RESULTS

The cgMLST scheme for *B. burgdorferi* s.l. contains 639 core loci

We have used 174 high-quality *B. burgdorferi* s.l. genomes of unique strains and 17 species (Tables 1 and S1; see STAR Methods for details) and 815 chromosomal coding sequences (CDS) of *B. burgdorferi* s.s. B31 from GenBank (GenBank: NC_001318.1/AE000783.1, November 15, 2023) to develop the cgMLST scheme.

The 174 genomes were analyzed for core loci that are present and designated in ≥95% of the genomes (with 97% identity over 99% alignment length). We identified 639 chromosomal core loci and included them in the *Borrelia* cgMLST scheme (see Table S2 for description and length information). A schematic overview of the cgMLST development is shown in Figure 1.

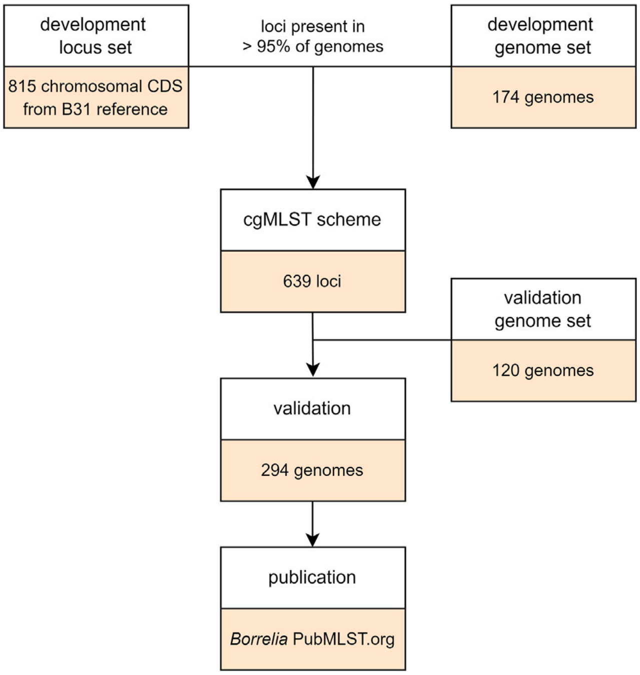

Figure 1. Schematic overview of the *B. burgdorferi* s.l. cgMLST scheme development

174 *B. burgdorferi* s.l. genomes were scanned for the presence of 815 chromosomal CDSs from B31 reference, resulting in 639 cgMLST loci. An additional 120 *B. burgdorferi* s.l. genomes were added to validate the scheme, resulting in 294 genomes.

cgMLST phylogeny has high statistical support and is not affected by recombination

Comparison of the unrooted maximum likelihood (ML) phylogenetic trees (n = 174 genomes) based on the 8-loci MLST vs. the developed 639-loci cgMLST scheme (Figures 2A and 2B, respectively) clearly showed that in both trees, samples clustered according to species and formed two major clades (an "American" and a "Eurasian" clade), as previously described.[35,36] A notable difference between MLST and cgMLST phylogenies was the clustering of *B. chilensis* and *B. maritima*.

In the MLST ML tree (Figure 2A) the American clade encompassed *B. americana*, *B. bissettiae*, *B. burgdorferi* s.s., *B. californiensis*, *B. carolinensis*, *B. kurtenbachii*, and *B. mayonii*. The Eurasian clade included *B. afzelii*, *B. bavariensis*, *B. garinii*, *B. japonica*, *B. spielmanii*, *B. turdi*, *B. valaisiana*, and *B. yangtzensis*. In this tree, the species *B. chilensis* and *B. maritima* (labeled in red in Figure 2A) clustered within the Eurasian clade as sister clades to *B. afzelii* and *B. spielmanii*. All of the internal nodes of the American clade were well supported (bootstrap values ≥ 95; colored yellow and green in Figure 2A). While some nodes of the Eurasian clade also showed high bootstrap values of 100 (colored green in Figure 2A), there were several internal nodes with lower bootstrap support (bootstrap values ranged from 67 to 87, colored red in Figure 2A, see the enlarged inset for details).

The phylogenetic tree based on the cgMLST scheme (Figure 2B) also showed clustering according to species, as well as the division into an American or Eurasian clade. However, the two strains belonging to the species *B. chilensis* and *B. maritima* (labeled in red in Figure 2B) did not cluster within the Eurasian clade; instead, they clustered between the Eurasian and American clades, appearing as sister clades to each other and to the Eurasian and American isolates, respectively. All internal nodes were well supported with bootstrap values of 100 (colored green in Figure 2B).

To analyze whether recombination events within cgMLST loci bias the phylogenetic reconstruction, we compared the cgMLST phylogeny to a phylogeny based on non-recombinant cgMLST regions. For this, the cgMLST alignment of the 174 development genomes was used to identify SNPs in non-recombinant regions. Based on these SNPs, an ML tree was generated (Figure 2C) and compared to the cgMLST ML tree (Figure 2B). Both trees showed the same clustering (according to species) and the same topology. The concordance of the cgMLST ML tree and the tree based on SNPs in non-recombinant regions of the cgMLST loci indicate that recombination does not affect the phylogeny.

To validate the scheme, a further 120 genomes were included, resulting in a total number of 294 genomes belonging to 17 *B. burgdorferi* s.l. species (see Tables 1 and S1 for detailed isolate information). The resulting cgMLST ML tree showed species-specific clustering in high resolution (high support of all internal nodes with bootstrap values of 100, colored green in Figure 2D; Figure 2D: unrooted ML tree; Figure 3: midpoint rooted circular ML tree). The cgMLST ML tree topology of 294 isolates (Figure 2D) is congruent with the topology of the cgMLST ML tree of the development genome set (174 isolates, Figure 2B), the division into an American or Eurasian clade, and *B. chilensis* and *B. maritima* clustering between the two major clades.

cgMLST improves lineage resolution within species

The distance matrices give information about the allelic differences between the isolates. For species for which at least two isolates were included in the validation, the minimum and maximum allelic differences between isolates of the same species are listed in Table 2. For species with more than five isolates (*B. afzelii*, *B. bavariensis*, *B. burgdorferi* s.s., *B. garinii*), the allelic differences ranged from 0 to 8 based on the 8-loci MLST scheme and from 0 to 637 allelic differences based on the 639-loci cgMLST scheme.

Based on the MLST and cgMLST distances matrices, minimum spanning trees (MSTs) were generated and visualized in GrapeTree (MLST: Figures 4A and 4B, cgMLST: Figures 4C and 4D). The MLST MSTs show clustering according to species (Figure 4A) and several isolates with identical MLST allele profiles clustered together with 0 AD (show as pie charts in Figures 4A and 4B), resulting in identical sequence type (ST) assignments (Figure 4B). In the cgMLST MSTs, isolates also clustered according to species (Figure 4C), but only two *B. afzelii*, two *B. bavariensis*, and two *B. garinii* isolates had the same cgMLST allele profile, while the other isolates had unique profiles, resulting in the assignment of different core genome STs (cgSTs) (Figures 4D and 3).

To group the cgSTs into a core genome cluster, single-linkage clustering was applied using thresholds of 100, 50, 25, 10, and 5 allelic differences. The cg clusters are designated with

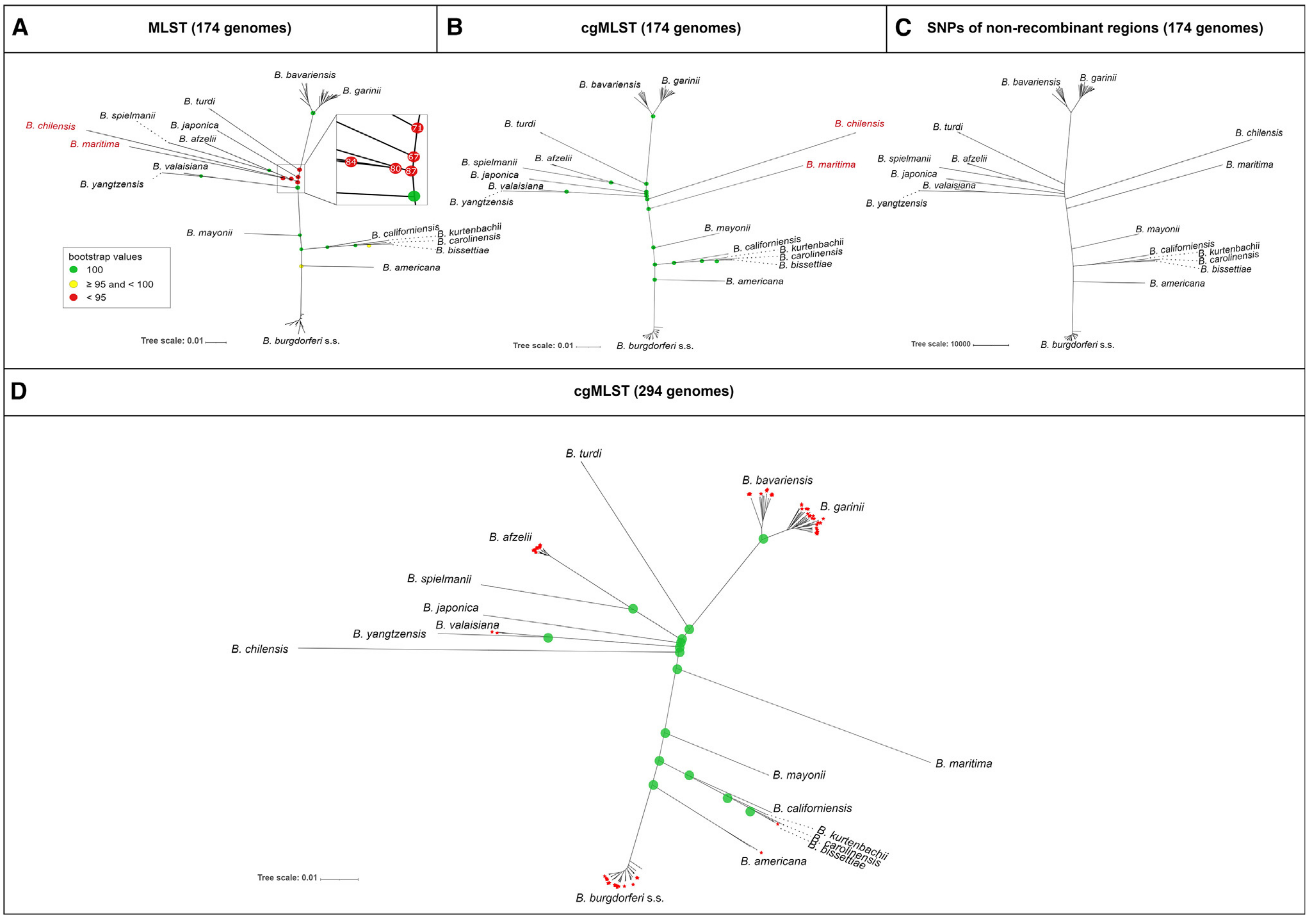

Figure 2. Unrooted ML trees of 174 and 294 genomes

Trees were generated with IQ-TREE 2.2.2.7[37] (A, B, and D) or RAxML v.8.2.12[38] (C). Isolates are labeled according to genospecies. The scale bars for (A), (B), and (D) denote substitutions per site, whereas the scale bar for (C) refers to SNPs.

(A) ML tree is based on 8-loci MLST of 174 isolates. Bootstrap values of internal nodes are shown in colored points (green: 100, yellow: <100 and ≥95, red: <95). The enlarged inset show details of bootstrap values <95.

(B) ML tree based on 639-loci cgMLST of 174 isolates. All internal nodes are colored green, indicating bootstrap values of 100.

(C) ML tree based on SNPs present in non-recombinant cgMLST regions of 174 isolates. No difference was found compared to the tree shown in (B).

(D) ML tree is based on 639-loci cgMLST of 294 isolates. The additional 120 samples are marked with red asterisks. All internal nodes are highly supported with bootstrap values of 100 (colored green).

identifier "Bb_cgc_" followed by the allelic difference thresholds used, e.g., Bb_cgc_100 for a threshold of 100 allelic differences. This resulted in 116 clusters of Bb_cgc_100 and up to 184 clusters of Bb_cgc_5. Table 3 shows the number of clusters depending on the applied allelic mismatch threshold.

Figures S2A–S2E show the MSTs colored according to the Bb_cgc clusters with the various thresholds (Figure S2A: Bb_cgc_100, Figure S2B: Bb_cgc_50, Figure S2C: Bb_cgc_25, Figure S2D: Bb_cgc_10, and Figure S2E: Bb_cgc_5).

Example: *B. bavariensis* European population

The species *B. bavariensis* is divided into two populations, a genetically heterogenic Asian population and a genetically homogeneous, almost clonal European population.[39,40] The clonal genetic characteristics of the European population are utilized to investigate how the new 639-loci cgMLST scheme impacts the differentiation of isolates of this closely related group.

The circular ML tree (Figure 3) shows the separation of *B. bavariensis* (colored red in the first inner circle of Figure 3) into an Asian and a European population (the branch of the European population is highlighted in yellow in Figure 3). The average nucleotide identity (ANI) values for the whole batch of *B. bavariensis* isolates ranged from 95.357 to 99.997, while values ranged from 96.431 to 99.997 for the heterogenic Asian and from 98.968 to 99.993 for the homogeneous European *B. bavariensis* isolates, respectively. ANI values for each pairwise comparison of the *B. bavariensis* isolates are shown in Table S3. The genetic characteristics can also be observed in the allelic differences of isolates within the two populations (Table 2). The Asian isolates (n = 30) show high variation in the MLST and cgMLST allelic differences, varying from 0 to 8 (MLST) and from 3 to 635 (cgMLST) (for details,

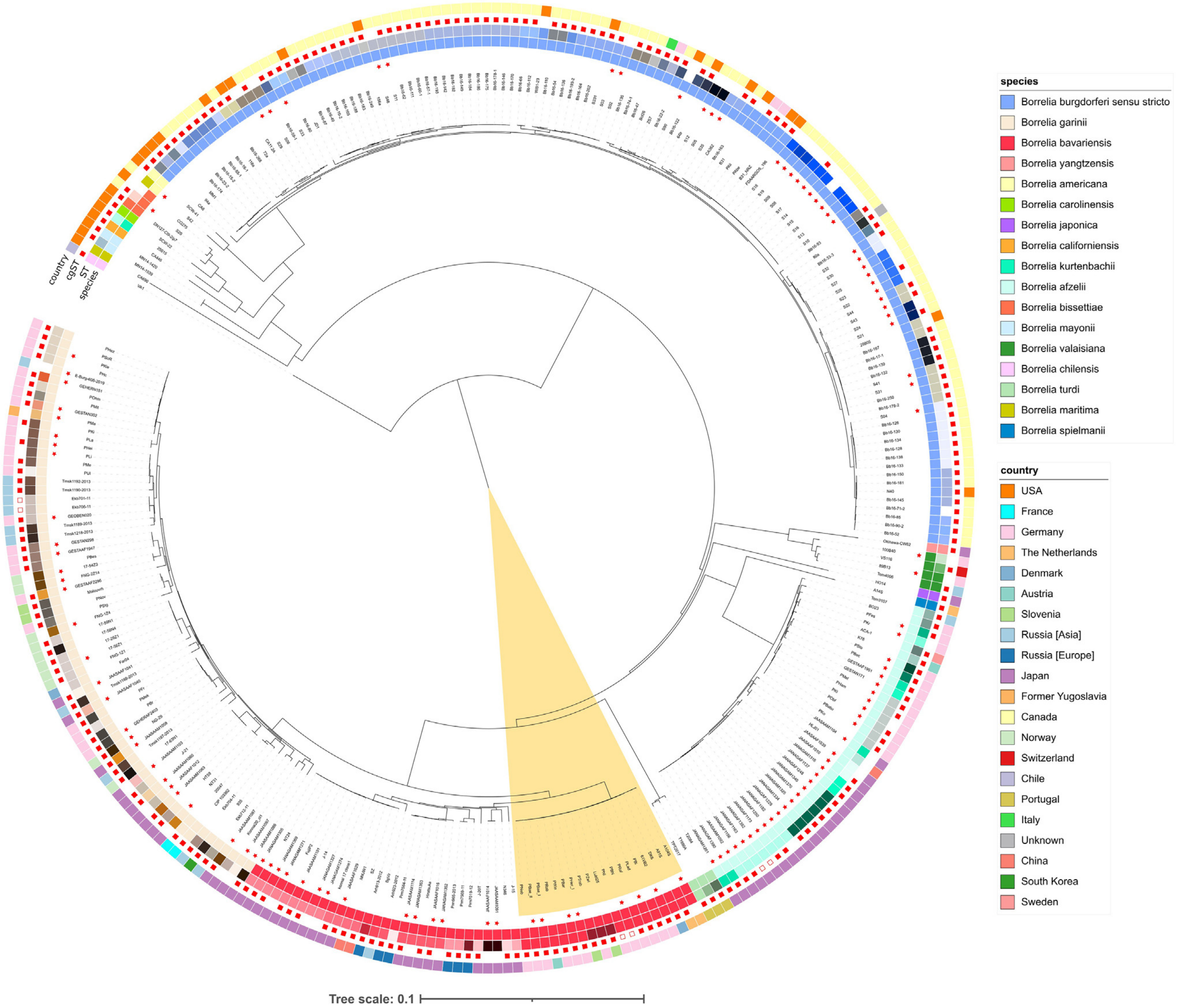

Figure 3. Midpoint rooted circular ML tree based on 639-loci cgMLST using the genome set of 294 isolates

Tree was generated with IQ-TREE 2.2.2.7.[37] The scale bar refers to nucleotide substitutions per site. Samples belonging to the validation genome set are marked with red asterisks. The different colors of the first inner circle represent the genospecies, and the second circle represents the ST (based on the 8-loci MLST scheme). The symbols in the third circle represent the cgST (based on the 639-loci cgMLST scheme): an empty field represents a missing value, unfilled red squares indicate that two samples of the same species share the same cgST, and filled red squares represent unique cgST values. The different colors in the outer circle represent the different countries of origin. The branch of the European *B. bavariensis* isolates is highlighted in yellow.

see Tables S4 and S5). While the homogeneous European isolates (n = 19) differ by a maximum of one MLST allele giving rise to just two STs (ST84 and ST85), the cgMLST scheme led to a higher resolution with a maximum of 179 allelic differences (for details, see Tables S6 and S7). One isolate (*B. bavariensis* A104S [*Borrelia* PubMLST: ID3756]) was not assigned to a cgST (>2% of loci were missing), and the other 18 isolates were assigned to 17 different cgSTs (two isolates had an identical cgST: *B. bavariensis* PBN [*Borrelia* PubMLST: ID3759] and *B. bavariensis* PNi [*Borrelia* PubMLST: ID2712]) (Figures 3 and 4D), showing that cgMLST has higher discriminatory power than MLST. Various allelic mismatch thresholds were applied to group the cgSTs (Figure S2, European *B. bavariensis* isolates are highlighted in yellow). Using a threshold of 100 allelic differences (meaning that isolates with cgMLST profiles differing in 100 loci or fewer will be assigned to the same Bb_cgc_100 cluster), the 18 European *B. bavariensis* isolates with assigned cgSTs shared the same Bb_cgc_100 cluster. By decreasing the threshold of allelic differences, the 18 isolates are separated into 4 (Bb_cgc_50), 5 (Bb_cgc_25), 6 (Bb_cgc_10), or 9 clusters (Bb_cgc_5).

Availability of the *B. burgdorferi* s.l. cgMLST scheme

The developed and validated 639-loci cgMLST scheme for *B. burgdorferi* s.l. is publicly available on the *Borrelia*

Table 2. Minimum and maximum ADs within isolates of the same genospecies based on the 8-loci MLST and 639-loci cgMLST schemes

		MLST		cgMLST	
Species	No. of isolates	Min. allelic differences	Max. allelic differences	Min. allelic differences	Max. allelic differences
B. afzelii	37	0	8	0	603
B. burgdorferi s.s.	120	0	8	1	616
B. garinii	66	0	8	0	637
B. bavariensis					
Total	49	0	8	0	637
Asian	30	0	8	3	635
European	19	0	1	0	179
B. valaisiana	4	0	7	331	591
B. bissettiae	3	6	8	492	594
B. turdi	3	4	8	483	492
B. americana	2	8	8	636	636
B. mayonii	2	1	1	5	5

B. bavariensis is additionally distinguished into isolates belonging to the Asian and European populations. ADs, allelic differences. See also Tables S4–S7.

PubMLST.org website (https://pubmlst.org/organisms/borrelia-spp).[17] Numerous analysis tools are listed in the STAR Methods.

DISCUSSION

Accurate whole-genome sequences have become available for many organisms. In order to build on the success of MLST and at the same time make use of the power of whole-genome data, we have developed a cgMLST scheme for *B. burgdorferi* s.l. While the 8-loci MLST scheme already enables unambiguous *Borrelia* genotyping,[12] we demonstrate here that the developed 639-loci cgMLST scheme has the same capabilities but with higher reliability and more discriminatory power.

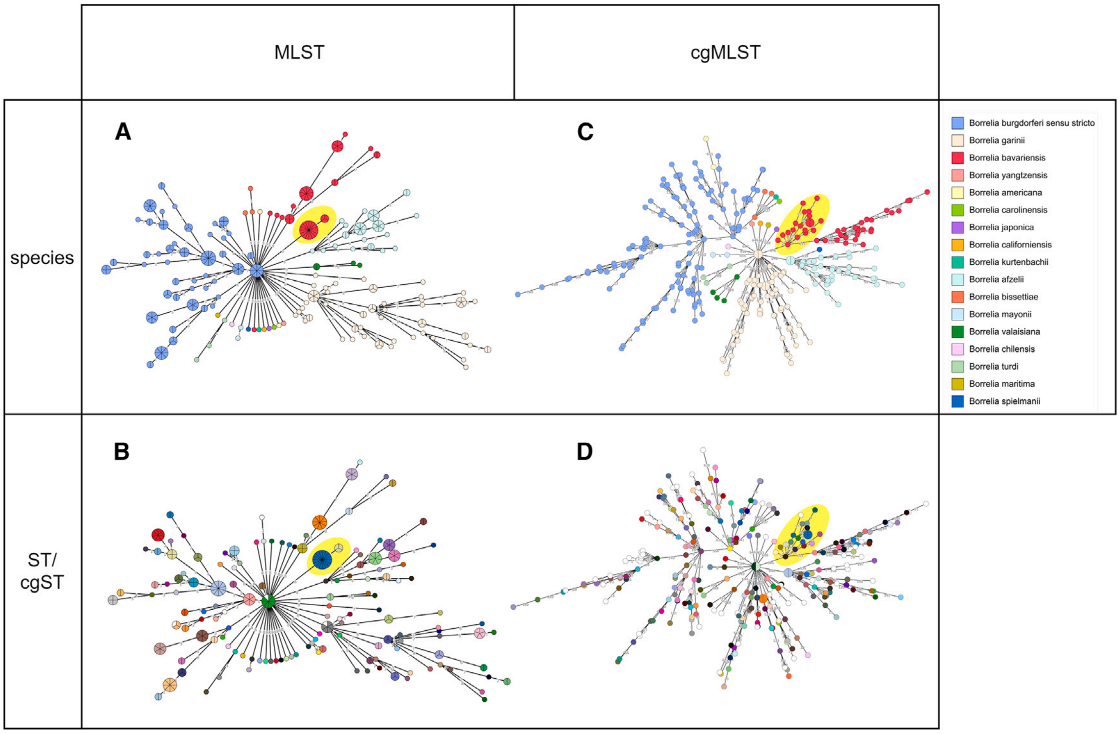

Figure 4. Minimum spanning trees of the 294 genomes.
The MSTs are based on the MLST (A and B) and cgMLST (C and D) typing schemes. The MSTs are colored according to species (A and C), ST (B), or cgST (D). Samples with missing STs (B) or cgSTs (D) are shown in white. The European *B. bavariensis* isolates are highlighted yellow.

Table 3. Number of core genome clusters (Bb_cgc) resulting from various thresholds

Core genome cluster name	Allelic mismatch threshold	No. of clusters
Bb_cgc_100	100	116
Bb_cgc_50	50	140
Bb_cgc_25	25	154
Bb_cgc_10	10	169
Bb_cgc_5	5	184

Phylogenetic analyses showed that 174 isolates clustered in both ML trees (8-loci MLST and 639-loci cgMLST) according to species and separated into an American (*B. americana*, *B. bissettiae*, *B. burgdorferi* s.s., *B. californiensis*, *B. carolinensis*, *B. kurtenbachii*, and *B. mayonii*) or Eurasian clade (*B. afzelii*, *B. bavariensis*, *B. garinii*, *B. japonica*, *B. spielmanii*, *B. turdi*, *B. valaisiana*, and *B. yangtzensis*) (with the caveat that *B. bissettiae* and *B. burgdorferi* s.s. also occur in Europe). Bootstraps values ranged from 67 to 100 in the MLST ML tree, and low bootstrap values (<95) are likely due to the limited number of analyzed loci. The developed cgMLST scheme used 639 core loci and provided a higher resolution and consistently well-supported internal nodes (bootstrap values of 100 for all inner nodes).

In previous studies based on the 8-loci MLST using 20 *B. burgdorferi* s.l. strains, *B. maritima* clustered within the Eurasian clade as a sister to *B. afzelii* and *B. spielmanii*,[41] which is in accordance with the 8-loci MLST results of this study. However, another study analyzing 37 single-copy genes of 114 *B. burgdorferi* s.l. strains and one relapsing fever species was in accordance with the data presented here using the 639-loci cgMLST, where *B. maritima* was a sister clade of the American clade.[35] High bootstrap values of internal nodes of 100 in the cgMLST phylogenies support the proposition of *B. maritima* being a sister clade of American species. Thus, it can be assumed that the cgMLST phylogeny represents a robust topology.

As recombination events can impact and bias phylogenetic reconstructions, we compared the phylogeny of the cgMLST ML tree and the ML tree based on SNPs of non-recombinant regions of the cgMLST alignment. As both ML trees showed the same clustering and topology, we suggest that recombination did not affect the phylogeny of the cgMLST ML tree. Evidence for chromosomal recombination has been suggested in previous studies on *B. burgdorferi* s.s. (the recombination-to-mutation ratio was >1 to 3)[42,43]; however, it did not affect the phylogenic analyses in those studies, tying in with our results presented here.[42,44]

Using a dataset of 294 genomes of the *B. burgdorferi* s.l. complex, phylogenetic analyses confirmed species-specific clustering of the cgMLST ML tree with consistently highly supported nodes (bootstrap values of 100 for all internal nodes). Furthermore, no difference in topology of the cgMLST ML trees (174 and 294 isolates) was noticed, with *B. chilensis* and *B. maritima* clustering in both trees between the Eurasian and the American clade. These data are in accordance with the phylogenetic analyses based on 37 single-copy genes conducted by Margos et al.[35] These results underscore the robustness of the developed 639-loci cgMLST typing scheme.

The cgMLST scheme also impacts the discriminatory power within species as exemplified using the species *B. bavariensis*. This species has genetically unique characteristics, as the it is divided into a heterogeneous Asian population and a homogeneous European population. The genetic bottleneck in the European populations is supposed to be the result of a vector switch (from *I. persulcatus* to *I. ricinus*), which enabled the species to invade Europe.[39,40] The genetic clonality of the European *B. bavariensis* isolates provided a suitable model to investigate whether the cgMLST scheme may allow the differentiation of strains of this population. Indeed, the 639-loci cgMLST scheme separated 18 isolates with assigned cgSTs into 17 different cgSTs. As expected, these data show that the cgMLST scheme enables population genetic analyses at an even finer scale than MLST.

As most of the isolates were assigned to a unique cgST, various allelic mismatch thresholds (100, 50, 25, 10, and 5) were used to group the cgSTs. We observed that the European *B. bavariensis* were grouped in the identical Bb_cgc_100 cluster (threshold: 100 allelic differences) but separated into different clusters based on stricter thresholds. This indicates that the threshold of 100 allelic differences may be a biologically meaningful threshold to group *Borrelia* isolates. It needs to be noted that the cg clusters are assigned group numbers that are not stable and therefore should not be used for nomenclature. Due to the single-linkage clustering, adding new genomes can lead to cg cluster merging, which in turn may lead to new group numbers.

In conclusion, we have extended the advantages of MLST to a genomic scale and have developed a 639-loci cgMLST scheme for *B. burgdorferi* s.l. that enables an improved insight into the evolutionary history of the species complex. Apart from unambiguous genotyping, the scheme allows fine-scale population structure analyses in high resolution and reliability even for genetically clonal samples as the European *B. bavariensis* population.

Limitations of the study

The 639-loci cgMLST scheme was established for whole-genome data and therefore requires cultivation of the bacteria. While the generation of sequence data is getting cheaper, faster, and, most importantly, more accurate, cultivating *Borrelia* is still fastidious, and thus MLST will still have a place in investigating environmental samples. The MLST scheme as well as the newly developed cgMLST scheme include conserved chromosomal genes and do not include plasmid genes, which may be under greater selective pressure than conserved chromosomal loci. Therefore, to address the question of the evolutionary relationship of *Borrelia*, conserved chromosomal loci are better suited as plasmid-encoded genes. Thus, all the analyses of this manuscript are only based on chromosomal loci and do not include any plasmid analyses. cgMLST enables improved insights in the evolutionary history of the species and strains and is a prerequisite to understand the biological meaning and/or clinical relevance of highly variable *Borrelia* plasmids, their presence, and their gene content. So far, the cgMLST scheme has been

developed and validated for species belonging to the *B. burgdorferi* s.l. complex, and further studies are required to test its suitability for relapsing fever- and reptile-associated *Borrelia* species. This will require the adoption or expansion of the scheme as necessary.

RESOURCE AVAILABILITY

Lead contact

Further information and requests for resources and reagents should be directed to and will be fulfilled by the lead contact, Sabrina Hepner (sabrina.hepner@lgl.bayern.de).

Materials availability

This study did not generate new unique reagents.

Data and code availability

- This paper analyzes existing, publicly available genome data of the *Borrelia* PubMLST database. The link to the database is listed in the key resources table. PubMLST ID information is listed in Table S1.
- This paper does not report any original code.
- Any additional information required to reanalyze the data reported in this paper is available from the lead contact upon request.

ACKNOWLEDGMENTS

The authors gratefully acknowledge Samuel K. Sheppard for his generous and expert support of this work. We would like to thank Nicholas H. Ogden, Shary Tyson, and Robert E. Rollins for providing the sequence data that were uploaded to the *Borrelia* PubMLST webpage. We would like to thank all members of the German National Reference Centre for *Borrelia* for technical support. The National Reference Center for *Borrelia* was funded by the Robert-Koch-Institut, Berlin. The sequencing was funded by the ESGBOR ESCMID Study Group. A.S., V.F., and G.M. are members of the ESGBOR ESCMID Study Group. PubMLST is funded by a Wellcome Trust Biomedical Resource Grant (218205/Z/19/Z).

AUTHOR CONTRIBUTIONS

Conceptualization, S.H., G.M., V.F., and K.A.J.; project administration, S.H., G.M., and V.F.; supervision, G.M., V.F., K.A.J., E.M., A.D., A.W., J.H., and A.S.; investigation, S.H., G.M., K.A.J., and S.C.-R.; formal analysis, S.H., G.M., K.A.J., and S.C.-R.; validation, S.H., G.M., K.A.J., and S.C.-R.; visualization, S.H.; data curation, S.H., G.M., E.M., and K.A.J.; writing – original draft, S.H. and G.M.; writing – review & editing, S.H., G.M., V.F., A.D., K.A.J., and E.M.

DECLARATION OF INTERESTS

The authors declare no competing interests.

STAR★METHODS

Detailed methods are provided in the online version of this paper and include the following:

- KEY RESOURCES TABLE
- METHODS DETAILS
  - Development of the cgMLST scheme
- QUANTIFICATION AND STATISTICAL ANALYSIS

SUPPLEMENTAL INFORMATION

Supplemental information can be found online at https://doi.org/10.1016/j.crmeth.2024.100935.

Received: July 18, 2024
Revised: September 6, 2024
Accepted: November 21, 2024
Published: December 18, 2024

REFERENCES

1. Stanek, G., Wormser, G.P., Gray, J., and Strle, F. (2012). Lyme borreliosis. Lancet *379*, 461–473. https://doi.org/10.1016/S0140-6736(11)60103-7.
2. Stanek, G., Fingerle, V., Hunfeld, K.P., Jaulhac, B., Kaiser, R., Krause, A., Kristoferitsch, W., O'Connell, S., Ornstein, K., Strle, F., and Gray, J. (2011). Lyme borreliosis: Clinical case definitions for diagnosis and management in Europe. Clin. Microbiol. Infect. *17*, 69–79.
3. Steere, A.C., Strle, F., Wormser, G.P., Hu, L.T., Branda, J.A., Hovius, J.W.R., Li, X., and Mead, P.S. (2016). Lyme borreliosis. Nat. Rev. Dis. Prim. *2*, 16090. https://doi.org/10.1038/nrdp.2016.90.
4. Margos, G., Wilske, B., Sing, A., Hizo-Teufel, C., Cao, W.C., Chu, C., Scholz, H., Straubinger, R.K., and Fingerle, V. (2013). *Borrelia bavariensis* sp. nov. is widely distributed in Europe and Asia. Int. J. Syst. Evol. Microbiol. *63*, 4284–4288.
5. Pritt, B.S., Respicio-Kingry, L.B., Sloan, L.M., Schriefer, M.E., Replogle, A.J., Bjork, J., Liu, G., Kingry, L.C., Mead, P.S., Neitzel, D.F., et al. (2016). *Borrelia mayonii* sp. nov., a member of the Borrelia burgdorferi sensu lato complex, detected in patients and ticks in the upper midwestern United States. Int. J. Syst. Evol. Microbiol. *66*, 4878–4880. https://doi.org/10.1099/ijsem.0.001445.
6. Margos, G., Henningsson, A.J., Hepner, S., Markowicz, M., Sing, A., and Fingerle, V. (2023). *Borrelia* ecology, evolution, and human disease: A mosaic of life. In Zoonoses: Infections Affecting Humans and Animals, A. Sing, ed. (Springer International Publishing), pp. 1–66. https://doi.org/10.1007/978-3-030-85877-3_49-1.
7. Margos, G., Hepner, S., and Fingerle, V. (2022). In Characteristics of *Borrelia burgdorferi* sensu lato, L. Borreliosis, K.-P. Hunfeld, and J. Gray, eds. (Springer International Publishing), pp. 1–29.
8. Fraser, C.M., Casjens, S., Huang, W.M., Sutton, G.G., Clayton, R., Lathigra, R., White, O., Ketchum, K.A., Dodson, R., Hickey, E.K., et al. (1997). Genomic sequence of a Lyme disease spirochaete, *Borrelia burgdorferi*. Nature *390*, 580–586.
9. Casjens, S. (2000). *Borrelia* genomes in the year 2000. J. Mol. Microbiol. Biotechnol. *2*, 401–410.
10. Casjens, S., Palmer, N., van Vugt, R., Huang, W.M., Stevenson, B., Rosa, P., Lathigra, R., Sutton, G., Peterson, J., Dodson, R.J., et al. (2000). A bacterial genome in flux: the twelve linear and nine circular extrachromosomal DNAs in an infectious isolate of the Lyme disease spirochete *Borrelia burgdorferi*. Mol. Microbiol. *35*, 490–516.
11. Wang, G., van Dam, A.P., Schwartz, I., and Dankert, J. (1999). Molecular typing of *Borrelia burgdorferi* sensu lato: taxonomic, epidemiological, and clinical implications. Clin. Microbiol. Rev. *12*, 633–653. https://doi.org/10.1128/cmr.12.4.633.
12. Margos, G., Gatewood, A.G., Aanensen, D.M., Hanincová, K., Terekhova, D., Vollmer, S.A., Cornet, M., Piesman, J., Donaghy, M., Bormane, A., et al. (2008). MLST of housekeeping genes captures geographic population structure and suggests a European origin of *Borrelia burgdorferi*. Proc. Natl. Acad. Sci. USA *105*, 8730–8735.
13. Maiden, M.C., Bygraves, J.A., Feil, E., Morelli, G., Russell, J.E., Urwin, R., Zhang, Q., Zhou, J., Zurth, K., Caugant, D.A., et al. (1998). Multilocus sequence typing: a portable approach to the identification of clones within populations of pathogenic microorganisms. Proc. Natl. Acad. Sci. USA *95*, 3140–3145.
14. Pérez-Losada, M., Cabezas, P., Castro-Nallar, E., and Crandall, K.A. (2013). Pathogen typing in the genomics era: MLST and the future of molecular epidemiology. Infect. Genet. Evol. *16*, 38–53. https://doi.org/10.1016/j.meegid.2013.01.009.

CellPress
OPEN ACCESS

15. Maiden, M.C.J., Jansen van Rensburg, M.J., Bray, J.E., Earle, S.G., Ford, S.A., Jolley, K.A., and McCarthy, N.D. (2013). MLST revisited: the gene-by-gene approach to bacterial genomics. Nat. Rev. Microbiol. *11*, 728–736. https://doi.org/10.1038/nrmicro3093.

16. Achtman, M., Wain, J., Weill, F.X., Nair, S., Zhou, Z., Sangal, V., Krauland, M.G., Hale, J.L., Harbottle, H., Uesbeck, A., et al. (2012). Multilocus sequence typing as a replacement for serotyping in *Salmonella enterica*. PLoS Pathog. *8*, e1002776.

17. Jolley, K.A., Bray, J.E., and Maiden, M.C.J. (2018). Open-access bacterial population genomics: BIGSdb software, the PubMLST.org website and their applications. Wellcome Open Res. *3*, 124. https://doi.org/10.12688/wellcomeopenres.14826.1.

18. Jolley, K.A., and Maiden, M.C.J. (2010). BIGSdb: Scalable analysis of bacterial genome variation at the population level. BMC Bioinf. *11*, 595. https://doi.org/10.1186/1471-2105-11-595.

19. Margos, G., Vollmer, S.A., Cornet, M., Garnier, M., Fingerle, V., Wilske, B., Bormane, A., Vitorino, L., Collares-Pereira, M., Drancourt, M., and Kurtenbach, K. (2009). A new *Borrelia* species defined by multilocus sequence analysis of housekeeping genes. Appl. Environ. Microbiol. *75*, 5410–5416.

20. Margos, G., Binder, K., Dzaferovic, E., Hizo-Teufel, C., Sing, A., Wildner, M., Fingerle, V., and Jolley, K.A. (2015). PubMLST.org - The new home for the *Borrelia* MLSA database. Ticks Tick. Borne. Dis. *6*, 869–871. https://doi.org/10.1016/j.ttbdis.2015.06.007.

21. Kneubehl, A.R., Krishnavajhala, A., Leal, S.M., Replogle, A.J., Kingry, L.C., Bermúdez, S.E., Labruna, M.B., and Lopez, J.E. (2022). Comparative genomics of the Western Hemisphere soft tick-borne relapsing fever borreliae highlights extensive plasmid diversity. BMC Genom. *23*, 410. https://doi.org/10.1186/s12864-022-08523-7.

22. Margos, G., Vollmer, S.A., Ogden, N.H., and Fish, D. (2011). Population genetics, taxonomy, phylogeny and evolution of *Borrelia burgdorferi* sensu lato. Infect. Genet. Evol. *11*, 1545–1563.

23. Bratcher, H.B., Corton, C., Jolley, K.A., Parkhill, J., and Maiden, M.C.J. (2014). A gene-by-gene population genomics platform: de novo assembly, annotation and genealogical analysis of 108 representative *Neisseria meningitidis* genomes. BMC Genom. *15*, 1138. https://doi.org/10.1186/1471-2164-15-1138.

24. Harrison, O.B., Cehovin, A., Skett, J., Jolley, K.A., Massari, P., Genco, C.A., Tang, C.M., and Maiden, M.C.J. (2020). *Neisseria gonorrhoeae* population genomics: Use of the gonococcal core genome to improve surveillance of antimicrobial resistance. J. Infect. Dis. *222*, 1816–1825. https://doi.org/10.1093/infdis/jiaa002.

25. Ruppitsch, W., Pietzka, A., Prior, K., Bletz, S., Fernandez, H.L., Allerberger, F., Harmsen, D., and Mellmann, A. (2015). Defining and evaluating a core genome multilocus sequence typing scheme for whole-genome sequence-based typing of *Listeria monocytogenes*. J. Clin. Microbiol. *53*, 2869–2876. https://doi.org/10.1128/jcm.01193-15.

26. Kohl, T.A., Diel, R., Harmsen, D., Rothgänger, J., Walter, K.M., Merker, M., Weniger, T., and Niemann, S. (2014). Whole-genome-based *Mycobacterium tuberculosis* surveillance: a standardized, portable, and expandable approach. J. Clin. Microbiol. *52*, 2479–2486. https://doi.org/10.1128/jcm.00567-14.

27. Leopold, S.R., Goering, R.V., Witten, A., Harmsen, D., and Mellmann, A. (2014). Bacterial whole-genome sequencing revisited: portable, scalable, and standardized analysis for typing and detection of virulence and antibiotic resistance genes. J. Clin. Microbiol. *52*, 2365–2370. https://doi.org/10.1128/jcm.00262-14.

28. Wang, Z., Gu, C., Sun, L., Zhao, F., Fu, Y., Di, L., Zhang, J., Zhuang, H., Jiang, S., Wang, H., et al. (2022). Development of a novel core genome MLST scheme for tracing multidrug resistant *Staphylococcus capitis*. Nat. Commun. *13*, 4254. https://doi.org/10.1038/s41467-022-31908-x.

29. Cody, A.J., Bray, J.E., Jolley, K.A., McCarthy, N.D., and Maiden, M.C.J. (2017). Core genome multilocus sequence typing scheme for stable, comparative analyses of *Campylobacter jejuni* and *C. coli* human disease isolates. J. Clin. Microbiol. *55*, 2086–2097. https://doi.org/10.1128/jcm.00080-17.

30. Alikhan, N.F., Zhou, Z., Sergeant, M.J., and Achtman, M. (2018). A genomic overview of the population structure of *Salmonella*. PLoS Genet. *14*, e1007261. https://doi.org/10.1371/journal.pgen.1007261.

31. Zhou, Z., Alikhan, N.F., Mohamed, K., Fan, Y., and Agama Study Group; and Achtman, M. (2020). The EnteroBase user's guide, with case studies on *Salmonella* transmissions, *Yersinia pestis* phylogeny, and *Escherichia* core genomic diversity. Genome Res. *30*, 138–152. https://doi.org/10.1101/gr.251678.119.

32. Pearce, M.E., Alikhan, N.F., Dallman, T.J., Zhou, Z., Grant, K., and Maiden, M.C.J. (2018). Comparative analysis of core genome MLST and SNP typing within a European *Salmonella* serovar Enteritidis outbreak. Int. J. Food Microbiol. *274*, 1–11. https://doi.org/10.1016/j.ijfoodmicro.2018.02.023.

33. Bialek-Davenet, S., Criscuolo, A., Ailloud, F., Passet, V., Jones, L., Delannoy-Vieillard, A.S., Garin, B., Le Hello, S., Arlet, G., Nicolas-Chanoine, M.H., et al. (2014). Genomic definition of hypervirulent and multidrug-resistant *Klebsiella pneumoniae* clonal groups. Emerg. Infect. Dis. *20*, 1812–1820. https://doi.org/10.3201/eid2011.140206.

34. Hennart, M., Guglielmini, J., Bridel, S., Maiden, M.C.J., Jolley, K.A., Criscuolo, A., and Brisse, S. (2022). A dual barcoding approach to bacterial strain nomenclature: Genomic taxonomy of *Klebsiella pneumoniae* strains. Mol. Biol. Evol. *39*, msac135. https://doi.org/10.1093/molbev/msac135.

35. Margos, G., Fedorova, N., Becker, N.S., Kleinjan, J.E., Marosevic, D., Krebs, S., Hui, L., Fingerle, V., and Lane, R.S. (2020). *Borrelia maritima* sp. nov., a novel species of the *Borrelia burgdorferi* sensu lato complex, occupying a basal position to North American species. Int. J. Syst. Evol. Microbiol. *70*, 849–856. https://doi.org/10.1099/ijsem.0.003833.

36. Becker, N.S., Margos, G., Blum, H., Krebs, S., Graf, A., Lane, R.S., Castillo-Ramírez, S., Sing, A., and Fingerle, V. (2016). Recurrent evolution of host and vector association in bacteria of the *Borrelia burgdorferi* sensu lato species complex. BMC Genom. *17*, 734. https://doi.org/10.1186/s12864-016-3016-4.

37. Nguyen, L.-T., Schmidt, H.A., von Haeseler, A., and Minh, B.Q. (2015). IQ-TREE: A fast and effective stochastic algorithm for estimating maximum-likelihood phylogenies. Mol. Biol. Evol. *32*, 268–274. https://doi.org/10.1093/molbev/msu300.

38. Stamatakis, A. (2014). RAxML version 8: a tool for phylogenetic analysis and post-analysis of large phylogenies. Bioinformatics *30*, 1312–1313. https://doi.org/10.1093/bioinformatics/btu033.

39. Gatzmann, F., Metzler, D., Krebs, S., Blum, H., Sing, A., Takano, A., Kawabata, H., Fingerle, V., Margos, G., and Becker, N.S. (2015). NGS population genetics analyses reveal divergent evolution of a Lyme Borreliosis agent in Europe and Asia. Ticks Tick. Borne. Dis. *6*, 344–351. https://doi.org/10.1016/j.ttbdis.2015.02.008.

40. Margos, G., Fingerle, V., and Reynolds, S. (2019). *Borrelia bavariensis*: Vector switch, niche invasion, and geographical spread of a tick-borne bacterial parasite. Front. Ecol. Evol. *7*, 401. https://doi.org/10.3389/fevo.2019.00401.

41. Fedorova, N., Kleinjan, J.E., James, D., Hui, L.T., Peeters, H., and Lane, R.S. (2014). Remarkable diversity of tick or mammalian-associated Borreliae in the metropolitan San Francisco Bay Area, California. Ticks Tick. Borne. Dis. *5*, 951–961. https://doi.org/10.1016/j.ttbdis.2014.07.015.

42. Tyler, S., Tyson, S., Dibernardo, A., Drebot, M., Feil, E.J., Graham, M., Knox, N.C., Lindsay, L.R., Margos, G., Mechai, S., et al. (2018). Whole genome sequencing and phylogenetic analysis of strains of the agent of Lyme disease *Borrelia burgdorferi* from Canadian emergence zones. Sci. Rep. *8*, 10552. https://doi.org/10.1038/s41598-018-28908-7.

43. Qiu, W.G., Schutzer, S.E., Bruno, J.F., Attie, O., Xu, Y., Dunn, J.J., Fraser, C.M., Casjens, S.R., and Luft, B.J. (2004). Genetic exchange and plasmid transfers in *Borrelia burgdorferi* sensu stricto revealed by three-way genome comparisons and multilocus sequence typing. Proc. Natl. Acad. Sci. USA *101*, 14150–14155.

44. Rollins, R.E., Sato, K., Nakao, M., Tawfeeq, M.T., Herrera-Mesías, F., Pereira, R.J., Kovalev, S., Margos, G., Fingerle, V., Kawabata, H., and Becker, N.S. (2023). Out of Asia? Expansion of Eurasian Lyme borreliosis causing genospecies display unique evolutionary trajectories. Mol. Ecol. *32*, 786–799. https://doi.org/10.1111/mec.16805.

45. Katoh, K., and Standley, D.M. (2013). MAFFT multiple sequence alignment software version 7: improvements in performance and usability. Mol. Biol. Evol. *30*, 772–780. https://doi.org/10.1093/molbev/mst010.

46. Hoang, D.T., Chernomor, O., von Haeseler, A., Minh, B.Q., and Vinh, L.S. (2018). UFBoot2: Improving the ultrafast bootstrap approximation. Mol. Biol. Evol. *35*, 518–522. https://doi.org/10.1093/molbev/msx281.

47. Kalyaanamoorthy, S., Minh, B.Q., Wong, T.K.F., von Haeseler, A., and Jermiin, L.S. (2017). ModelFinder: fast model selection for accurate phylogenetic estimates. Nat. Methods *14*, 587–589. https://doi.org/10.1038/nmeth.4285.

48. Letunic, I., and Bork, P. (2021). Interactive Tree Of Life (iTOL) v5: an online tool for phylogenetic tree display and annotation. Nucleic Acids Res. *49*, W293–W296. https://doi.org/10.1093/nar/gkab301.

49. Croucher, N.J., Page, A.J., Connor, T.R., Delaney, A.J., Keane, J.A., Bentley, S.D., Parkhill, J., and Harris, S.R. (2015). Rapid phylogenetic analysis of large samples of recombinant bacterial whole genome sequences using Gubbins. Nucleic Acids Res. *43*, e15. https://doi.org/10.1093/nar/gku1196.

50. Simonsen, M., Mailund, T., and Pedersen, C.N. (2008). In Rapid neighbour-joining. held in Karlsruhe, Germany, K.A. Crandall and J. Lagergren, eds. (Springer), pp. 113–122.

51. Zhou, Z., Alikhan, N.F., Sergeant, M.J., Luhmann, N., Vaz, C., Francisco, A.P., Carriço, J.A., and Achtman, M. (2018). GrapeTree: visualization of core genomic relationships among 100,000 bacterial pathogens. Genome Res. *28*, 1395–1404. https://doi.org/10.1101/gr.232397.117.

52. Pritchard, L., Glover, R.H., Humphris, S., Elphinstone, J.G., and Toth, I.K. (2016). Genomics and taxonomy in diagnostics for food security: soft-rotting enterobacterial plant pathogens. Anal. Methods *8*, 12–24. https://doi.org/10.1039/C5AY02550H.

53. Margos, G., Hepner, S., Mang, C., Marosevic, D., Reynolds, S.E., Krebs, S., Sing, A., Derdakova, M., Reiter, M.A., and Fingerle, V. (2017). Lost in plasmids: next generation sequencing and the complex genome of the tick-borne pathogen *Borrelia burgdorferi*. BMC Genom. *18*, 422. https://doi.org/10.1186/s12864-017-3804-5.

54. Casjens, S.R., Mongodin, E.F., Qiu, W.G., Luft, B.J., Schutzer, S.E., Gilcrease, E.B., Huang, W.M., Vujadinovic, M., Aron, J.K., Vargas, L.C., et al. (2012). Genome stability of Lyme disease spirochetes: comparative genomics of *Borrelia burgdorferi* plasmids. PLoS One *7*, e33280. https://doi.org/10.1371/journal.pone.0033280.

55. Casjens, S.R., Gilcrease, E.B., Vujadinovic, M., Mongodin, E.F., Luft, B.J., Schutzer, S.E., Fraser, C.M., and Qiu, W.G. (2017). Plasmid diversity and phylogenetic consistency in the Lyme disease agent *Borrelia burgdorferi*. BMC Genom. *18*, 165. https://doi.org/10.1186/s12864-017-3553-5.

56. Casjens, S.R., Di, L., Akther, S., Mongodin, E.F., Luft, B.J., Schutzer, S.E., Fraser, C.M., and Qiu, W.G. (2018). Primordial origin and diversification of plasmids in Lyme disease agent bacteria. BMC Genom. *19*, 218. https://doi.org/10.1186/s12864-018-4597-x.

57. Mongodin, E.F., Casjens, S.R., Bruno, J.F., Xu, Y., Drabek, E.F., Riley, D.R., Cantarel, B.L., Pagan, P.E., Hernandez, Y.A., Vargas, L.C., et al. (2013). Inter- and intra-specific pan-genomes of *Borrelia burgdorferi* sensu lato: genome stability and adaptive radiation. BMC Genom. *14*, 693. https://doi.org/10.1186/1471-2164-14-693.

58. Francisco, A.P., Vaz, C., Monteiro, P.T., Melo-Cristino, J., Ramirez, M., and Carriço, J.A. (2012). PHYLOViZ: phylogenetic inference and data visualization for sequence based typing methods. BMC Bioinf. *13*, 87. https://doi.org/10.1186/1471-2105-13-87.

59. Argimon, S., Abudahab, K., Goater, R.J.E., Fedosejev, A., Bhai, J., Glasner, C., Feil, E.J., Holden, M.T.G., Yeats, C.A., Grundmann, H., et al. (2016). Microreact: visualizing and sharing data for genomic epidemiology and phylogeography. Microb. Genom. *2*, e000093. https://doi.org/10.1099/mgen.0.000093.

STAR★METHODS

KEY RESOURCES TABLE

REAGENT or RESOURCE	SOURCE	IDENTIFIER
Deposited data		
B. burgdorferi s.s. B31 genome data (extraction of chromosomal coding sequences; extraction date: November 15, 2023)	GenBank	NC_001318.1/AE000783.1
Genome set (development and validation of cgMLST scheme, see Table S1 for PubMLST id information)	*Borrelia* PubMLST database	https://pubmlst.org/organisms/borrelia-spp
Software and algorithms		
BIGSdb software	Jolley et al.[17]	https://github.com/kjolley/BIGSdb
MAFFT (used for "Sequence Export" BIGSdb tool)	Katoh et al.[45]	https://mafft.cbrc.jp/alignment/software/linuxportable.html
IQ-TREE v2.2.2.7	Nguyen et al.[37] Hoang et al.[46] Kalyaanamoorthy et al.[47]	https://github.com/iqtree/iqtree2
iTOL v6.8.1	Letunic et al.[48]	https://itol.embl.de/
Gubbins v3.2.1	Croucher et al.[49]	https://github.com/nickjcroucher/gubbins
rapidnj v2.3.2	Simonsen et al.[50]	https://github.com/somme89/rapidNJ
RAxML v8.2.12	Stamatakis[38]	https://github.com/stamatak/standard-RAxML
GrapeTree (built-in BIGSdb)	Zhou et al.[51]	https://achtman-lab.github.io/GrapeTree/MSTree_holder.html
PYANI v0.2.12	Pritchard et al.[52]	https://github.com/widdowquinn/pyani

METHODS DETAILS

Development of the cgMLST scheme

Loci set

For the development of the scheme chromosomal coding sequences (CDS) (n = 815) of *B. burgdorferi* s.s. B31 from GenBank: NC_001318.1/AE000783.1 were extracted (extraction date: November 15, 2023) and defined in BIGSdb using built-in functionality. Unique identifiers in the format BORRxxxx were assigned with original designations defined as aliases. This independent locus nomenclature allows for the future addition of new loci not present in *B. burgdorferi* s.s. B31.

Genome set

High quality genome assemblies (see criteria below) of unique strains and available through the *Borrelia* PubMLST website (December 28, 2023, Table S1) were used as the development genome set (n = 174). Published results have shown that most genes on the chromosome are core genes.[10,53–57] Insufficient DNA quality and low coverage can result in low quality genomes that are missing a noticeably high number of loci (unpublished data). To identify high and low quality genomes all available genomes from the *Borrelia* PubMLST database (December 28, 2023; 177 genomes belonging to 17 *B burgdorferi* s.l. species, Table S1) were scanned against the 815 chromosomal B31 CDS using the BIGSdb "Gene Presence" analysis tool with default settings (70% min identity, 50% min alignment, BLASTN word size of 20). Our analyses showed that 175 genomes were missing <4%, which were considered high quality genomes. The remaining two genomes (*B. bavariensis* Tmsk976-2013 [*Borrelia* PubMLST: ID2722] and *B. garinii* Tmsk1193-2013 [*Borrelia* PubMLST: ID2751]) missed >14% of the loci and thus, were considered low quality genomes and were excluded from the genome set. Two of the 175 high quality genomes were identified as duplicates (*B. garinii* PBr; *Borrelia* PubMLST: ID2723 and ID2733) of which one (*Borrelia* PubMLST: ID2733) was excluded from the dataset. Thus, the development genome comprised 174 genomes and included samples of 17 *B burgdorferi* s.l. species (Table 1, see Table S1 for detailed isolate information).

cgMLST scheme set up and refinement

The 174 genomes were annotated at the defined loci using successive rounds of automated BIGSdb allele assignment using thresholds of 97% identity and over 99% alignment length compared to the closest matching allele. Auto-assigned alleles were complete coding sequences with consensus start codons, no internal stop codons, and an in-frame stop codon. Some manual curation was

also necessary to assign exemplary alleles that were slightly different lengths to those found in B31. Loci that were present and designated in $\geq$ 95% of the genomes were included in the cgMLST scheme.

Core genome sequence types and clustering

Core genome sequence types (cgSTs) were assigned for samples with $\leq$ 2% missing loci (13 loci). Missing cgMLST loci were assigned as "N" in profiles and ignored in pairwise comparisons. This can result in some isolates potentially having more than one cgST where profiles are identical apart from the presence of missing loci (see example in Figure S1). To group the cgSTs, single linkage clustering was applied using various allelic mismatch thresholds (100, 50, 25, 10, and 5 loci). Core genome clusters were designated with "Bb_cgc" indicating *B. burgdorferi* s.l. core genome cluster followed by the allelic mismatch threshold used, although it should be noted that these cluster groups are not stable due to merging as data are added and therefore should not be used for nomenclature.

Phylogenetic and GrapeTree analyses

Phylogenetic analyses based on the MLST scheme and the developed cgMLST scheme were performed with the 174 genomes and results were compared. A MAFFT[45] alignment of the concatenated MLST and cgMLST sequences was generated on the *Borrelia* PubMLST.org Website using the "Sequence Export" tool and the options "MAFFT aligner"[45] and "concatenate in frame". Maximum likelihood (ML) trees were generated with IQ-TREE 2.2.2.7,[37] 1000 ultrafast bootstrap replicons (UFBoot)[46] using the substitution models GTR+F+I+R3 for MLST and GTR+F+I+R6 for cgMLST phylogenies, respectively. ModelFinder as part of IQ-TREE[47] was used for model selection. The ML trees were visualized and edited using the online tool iTOL (Interactive Tree of Life) v6.8.1.[48]

To analyze if recombination in the cgMLST loci affected the phylogeny, the cgMLST ML tree was compared to an ML tree based on SNPs found in non-recombinant regions of the cgMLST loci. A phylogeny without recombination was constructed using Gubbins v3.2.1.[49] For the first phylogeny rapidnj v2.3.2[50] (model: JC) was used, followed by a subsequent iteration of an ML tree using RAxML v8.2.12[38] (model: GTRGAMMA) and 7 iterations.

An additional 120 genomes (validation genome set, Tables 1 and S1), uploaded to the *Borrelia* PubMLST website between December 28, 2023 and March 25, 2024, were included in the analyses (294 genomes in total). For phylogenetic analyses, a MAFFT[45] alignment of the concatenated cgMLST loci was generated by the built-in BIGSdb iTOL tool and uploaded to the iTOL website.[48] As before, the ML tree was generated with IQ-TREE 2.2.2.7[37] using same settings.

Distance matrices based on the MLST or cgMLST scheme were generated using BIGSdb "Genome Comparator" with default settings (70% min identity, 50% min alignment, BLASTN word size of 20, pairwise ignoring missing values). The output also included information about the sequence type (ST) (based on MLST) and the cgST (based on cgMLST). GrapeTree[51] was used to generate and visualize a minimum spanning tree (MST) using the built-in BIGSdb GrapeTree tool and selecting the MLST or cgMLST scheme.

Average nucleotide identity (ANI) analyses

The ANI values between *B. bavariensis* isolates were calculated using PYANI v0.2.12[52] choosing BLAST+ method (ANIb) to align 1020 nt fragments of the input sequences.

Example analysis tools available on the Borrelia PubMLST.org website

(1) Sequence Export: enables the download of loci sequences and also of the MAFFT[45] alignment of the concatenated scheme loci of up to 200 isolates from the database.
(2) Third party analyses tools: such as GrapeTree,[51] PhyloViz[58] (generating minimum spanning trees and distance matrices), iTOL[48] and Microreact[59] (generating neighbor joining trees and the MAFFT[45] alignment of the concatenated scheme loci).
(3) Genome Comparator: generates distance matrices, alignment of the concatenated scheme loci, splits graph and generates several further outputs. The tool provides the possibility to include external users' genomes that are currently under investigation but not published yet.

Detailed information about the website and their applications can be found in Jolley et al.[17]

QUANTIFICATION AND STATISTICAL ANALYSIS

For the validation of the developed cgMLST scheme phylogenetic, GrapeTree and ANI analyses were conducted. For the phylogenetic analyses ML trees were generated using IQ-TREE 2.2.2.7,[37] 1000 ultrafast bootstrap replicons (UFBoot)[46] and ModelFinder as part of IQ-TREE[47] for model selection. In order to evaluate the statistical support of inner nodes, the bootstrap values were displayed as colored points in the ML trees (green: 100, yellow: <100 and $\geq$ 95, red: <95). Additionally, phylogeny without recombination was constructed using Gubbins v3.2.1,[49] rapidnj v2.3.2[50] and RAxML v8.2.12.[38] For the ML trees visualization and editing the online tool iTOL v6.8.1[48] was used. The BIGSdb "Genome Comparator" was used to generate distance matrices and the built-in BIGSdb GrapeTree[51] tool was used to generate and visualize the MSTs based on the distance matrices. The ANI values were calculated using PYANI v0.2.12.[52] Additional details are provided in the method details section, results and figure legends.

Cell Reports Methods, Volume *5*

Supplemental information

A core genome MLST scheme for *Borrelia burgdorferi* sensu lato improves insights into the evolutionary history of the species complex

Sabrina Hepner, Keith A. Jolley, Santiago Castillo-Ramirez, Evangelos Mourkas, Alexandra Dangel, Andreas Wieser, Johannes Hübner, Andreas Sing, Volker Fingerle, and Gabriele Margos

Supplementary information

Supplemental figures

Figure S1: Example of the assignment of the core genome sequence type (cgST) and clustering to core genome clusters (Bb_cgc), related to STAR Methods.

Figure S2: Minimum spanning tree based on the cgMLST scheme using 294 isolates, related to Table 3 and Figure 4C-D.

Supplemental tables

Table S4: Distance matrix of the Asian *B. bavariensis* isolates (n=30) based on MLST, related to Table 2 and Figure 4.

Table S5: Distance matrix of the Asian *B. bavariensis* isolates (n=30) based on cgMLST, related to Table 2 and Figure 4.

Table S6: Distance matrix of the European *B. bavariensis* isolates (n=19) based on MLST, related to Table 2 and Figure 4.

Table S7: Distance matrix of the European *B. bavariensis* isolates (n=19) based on cgMLST, related to Table 2 and Figure 4.

Supplemental figures

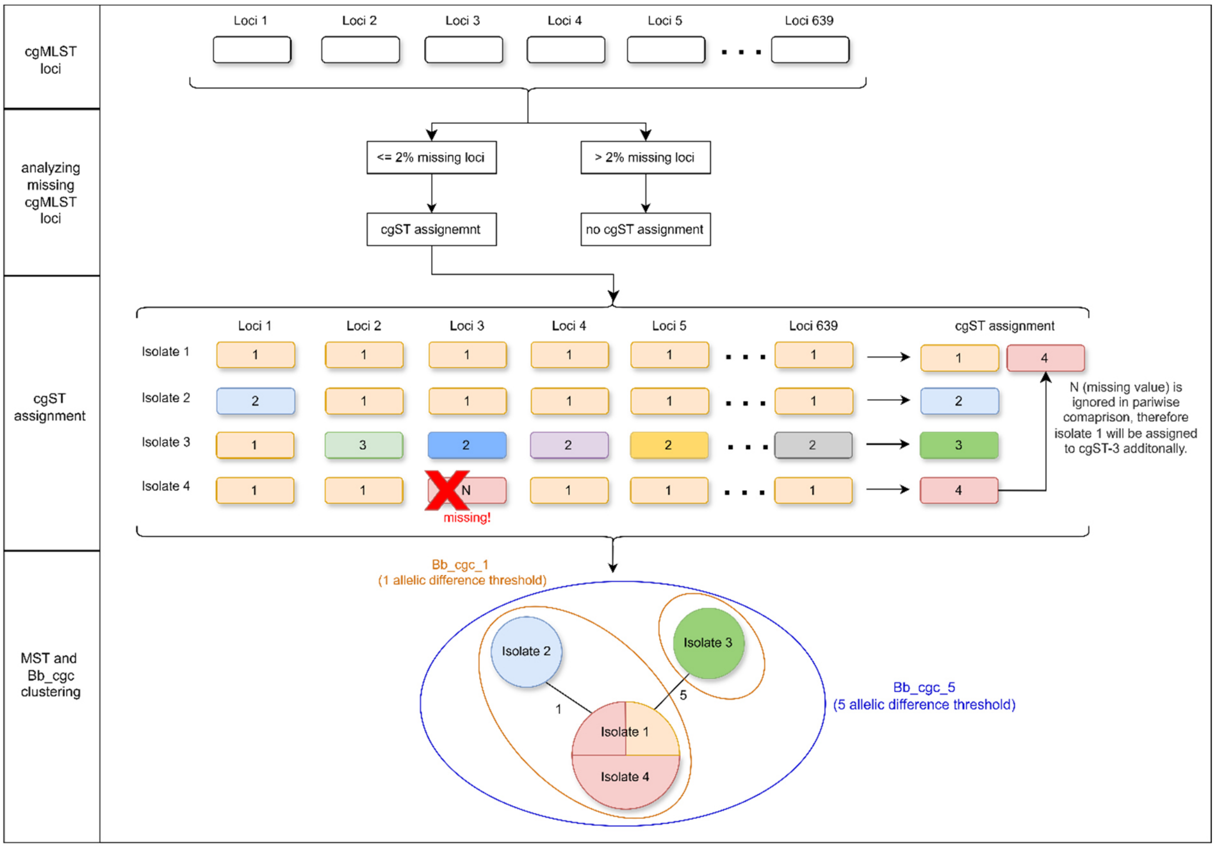

Figure S1: Example of the assignment of the core genome sequence type (cgST) and clustering to core genome clusters (Bb_cgc), related to STAR Methods.

In isolate 1, 2, 3 and 4 <= 2% of the cgMLST loci were missing resulting in cgST assignment. Isolate 1 with a new unique allelic profile (no missing values) is assigned to cgST-1. Isolate 2 differs in loci 1 and therefore has a new unique profile and is assigned to cgST-2. Isolate 3 has a new unique allelic profile and is assigned to cgST-3. Isolate 4 has the identical allele profile as isolate 1, but loci 3 is missing and therefore will be assigned with “N”. As it is not known which allele is missing, a new cgST-4 will be assigned. As missing values (“N”) will be ignored in pairwise comparison, isolate 1 will also match cgST-4 and also receive this designation. Comparing the isolates regarding allelic differences, isolate 1 and isolate 4 has 0 allelic differences, while these two isolates has one allelic difference to isolate 2 and five allelic differences to isolate 3 which can be visualized in the MST. Applying a threshold of five allelic differences, all isolates belong the same Bb_cgc_5 cluster, while they fell in two different Bb_cgc_1 clusters using a threshold of one allelic difference.

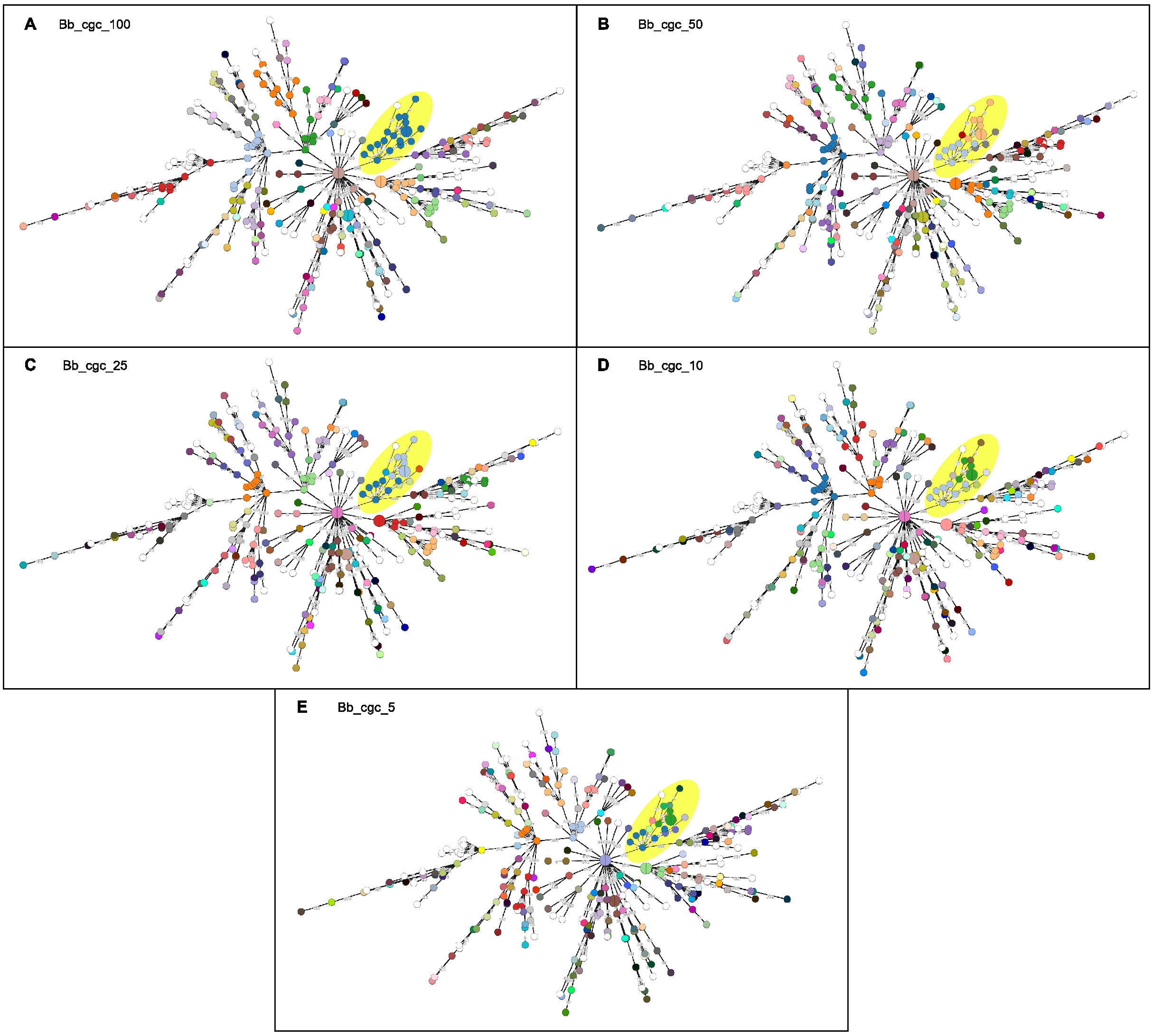

Figure S2: Minimum spanning tree based on the cgMLST scheme using 294 isolates, related to Table 3 and Figure 4C-D.

MST are colored according to *B. burgdorferi* core genome cluster (Bb_cgc) applying various allelic differences thresholds: (A) Bb_cgc_100, (B) Bb_cgc_50, (C) Bb_cgc_25, (D) Bb_cgc_10, (E) and Bb_cgc_5. Isolates with missing cgST are shown in white.

Supplemental tables

Table S4: Distance matrix of the Asian *B. bavariensis* isolates (n=30) based on MLST, related to Table 2 and Figure 4.

Arh913-2012_wgs																														
Arh923-2012_wgs	8																													
FujiP2_wgs	3	7																												
Hiratsuka_wgs	8	0	7																											
J-14_wgs	3	7	0	7																										
J-15_wgs	8	8	8	8	8																									
J-20T_wgs	8	8	8	8	8	8																								
konnai_17_clone1_wgs	1	8	3	8	3	8	8																							
N346_wgs	8	8	8	8	8	8	8	8																						
Prm965-2013_wgs	8	7	8	7	8	8	8	8	8																					
Prm7019-12_wgs	8	8	8	8	8	8	8	8	8	7																				
Prm7564-11_wgs	8	1	8	1	8	8	8	8	8	7	8																			
Prm7569-11_wgs	8	7	8	7	8	8	8	8	8	0	7	7																		
BgVir_wgs	8	8	8	8	8	8	8	8	8	8	8	8	8																	
NMJW1_wgs	1	8	3	8	3	8	8	0	8	8	8	8	8	8																
SZ_wgs	1	8	3	8	3	8	8	0	8	8	8	8	8	8	0															
JAASAAF1014_wgs	8	8	8	8	8	8	8	8	8	7	7	8	7	8	8	8														
JAASAAF1016_wgs	8	0	7	0	7	8	8	8	8	7	8	1	7	8	8	8	8													
JAASAAF1029_wgs	1	8	3	8	3	8	8	0	8	8	8	8	8	8	0	0	8	8												
JAASAAM1091_wgs	8	8	8	8	8	8	8	8	8	7	7	8	7	8	8	8	0	8	8											
JAASAAM1101_wgs	3	7	0	7	0	8	8	3	8	8	8	8	8	8	3	3	8	7	3	8										
JAASAAM1114_wgs	8	0	7	0	7	8	8	8	8	7	8	1	7	8	8	8	8	0	8	8	7									
JANAGAM1271_wgs	3	7	0	7	0	8	8	3	8	8	8	8	8	8	3	3	8	7	3	8	0	7								
JANAGAM1274_wgs	3	7	0	7	0	8	8	3	8	8	8	8	8	8	3	3	8	7	3	8	0	7	0							
JANAGAM1305_wgs	3	7	0	7	0	8	8	3	8	8	8	8	8	8	3	3	8	7	3	8	0	7	0	0						
JANAGAM1327_wgs	3	7	0	7	0	8	8	3	8	8	8	8	8	8	3	3	8	7	3	8	0	7	0	0	0					
JANAGAM1352_wgs	8	0	7	0	7	8	8	8	8	7	8	1	7	8	8	8	8	0	8	8	7	0	7	7	7	7				
JANAGAM1353_wgs	8	0	7	0	7	8	8	8	8	7	8	1	7	8	8	8	8	0	8	8	7	0	7	7	7	7	0			
JANAGAM1369_wgs	3	7	0	7	0	8	8	3	8	8	8	8	8	8	3	3	8	7	3	8	0	7	0	0	0	0	7	7		
NT24_wgs	3	7	0	7	0	8	8	3	8	8	8	8	8	8	3	3	8	7	3	8	0	7	0	0	0	0	7	7	0	

Table S5: Distance matrix of the Asian *B. bavariensis* isolates (n=30) based on cgMLST, related to Table 2 and Figure 4.

Arh913-2012_wgs																														
Arh923-2012_wgs	624																													
FujiP2_wgs	159	624																												
Hiratsuka_wgs	626	177	626																											
J-14_wgs	160	624	12	626																										
J-15_wgs	630	635	631	635	631																									
J-20T_wgs	632	634	631	634	631	632																								
konnai_17_clone1_wgs	98	623	155	625	155	630	631																							
N346_wgs	631	630	633	630	633	631	633	631																						
Prm965-2013_wgs	626	623	626	624	626	633	631	626	633																					
Prm7019-12_wgs	629	626	628	628	628	633	632	628	632	555																				
Prm7564-11_wgs	624	25	624	180	624	635	634	623	630	623	626																			
Prm7569-11_wgs	625	624	625	626	625	633	630	625	632	61	558	624																		
BgVir_wgs	608	623	610	625	609	632	632	607	628	625	625	623	624																	
NMJW1_wgs	98	623	149	625	149	630	631	43	631	626	628	623	625	607																
SZ_wgs	96	623	147	625	147	630	631	40	631	626	628	623	625	607	3															
JAASAAF1014_wgs	626	630	627	631	627	630	634	626	629	630	633	630	631	630	626	626														
JAASAAF1016_wgs	625	181	625	70	625	635	634	624	630	624	627	183	625	625	624	624	628													
JAASAAF1029_wgs	94	624	150	626	150	630	631	23	631	627	628	624	626	607	42	39	626	625												
JAASAAM1091_wgs	626	630	627	631	627	629	634	626	628	629	632	630	630	630	626	626	16	628	626											
JAASAAM1101_wgs	168	624	25	626	25	631	631	163	633	626	628	624	625	610	157	155	627	625	159	627										
JAASAAM1114_wgs	624	194	624	119	624	635	634	623	629	624	627	196	625	624	623	623	628	109	624	628	624									
JANAGAM1271_wgs	177	625	35	627	35	631	631	172	633	627	628	625	626	609	165	165	627	626	166	627	46	624								
JANAGAM1274_wgs	242	625	122	627	123	632	631	235	633	627	629	625	626	613	232	231	628	625	235	628	131	625	137							
JANAGAM1305_wgs	166	624	19	626	19	631	631	160	633	626	628	624	625	609	154	152	627	624	156	627	30	623	41	129						
JANAGAM1327_wgs	176	624	34	626	27	631	631	171	633	626	628	624	625	609	167	165	626	624	166	626	46	621	53	133	41					
JANAGAM1352_wgs	619	330	621	268	620	634	635	618	631	627	629	334	627	626	618	618	624	235	620	624	619	290	619	620	619	613				
JANAGAM1353_wgs	624	199	624	129	624	635	634	623	629	623	627	200	625	624	623	623	626	119	624	626	624	65	621	624	623	622	296			
JANAGAM1369_wgs	165	624	18	626	18	631	631	159	633	626	628	624	625	609	153	151	627	624	154	627	29	623	40	129	7	38	619	623		
NT24_wgs	166	624	20	626	20	631	631	159	633	626	629	624	625	609	154	152	627	624	156	627	31	623	42	130	6	42	619	623	8	

Table S6: Distance matrix of the European *B. bavariensis* isolates (n=19) based on MLST, related to Table 2 and Figure 4.

PBi																			
A91S_wgs	0																		
DK6_wgs	0	0																	
Lubl25_wgs	1	1	1																
PNeb_wgs	0	0	0	1															
PNi_wgs	0	0	0	1	0														
PRab_wgs	0	0	0	1	0	0													
PRof_wgs	0	0	0	1	0	0	0												
PTrob_wgs	1	1	1	0	1	1	1	1											
PWin_wgs	0	0	0	1	0	0	0	0	1										
PZwi_wgs	1	1	1	0	1	1	1	1	0	1									
61VB2_wgs	0	0	0	1	0	0	0	0	1	0	1								
PBae_I_wgs	0	0	0	1	0	0	0	0	1	0	1	0							
A104S_wgs	0	0	0	1	0	0	0	0	1	0	1	0	0						
PBae_II_wgs	0	0	0	1	0	0	0	0	1	0	1	0	0	0					
PBar_wgs	0	0	0	1	0	0	0	0	1	0	1	0	0	0	0				
PBN_wgs	0	0	0	1	0	0	0	0	1	0	1	0	0	0	0	0			
PHer_I_wgs	0	0	0	1	0	0	0	0	1	0	1	0	0	0	0	0	0		
PLad_wgs	0	0	0	1	0	0	0	0	1	0	1	0	0	0	0	0	0	0	

Table S7: Distance matrix of the European *B. bavariensis* isolates (n=19) based on cgMLST, related to Table 2 and Figure 4.

PBi																			
A91S_wgs	107																		
DK6_wgs	12	104																	
Lubl25_wgs	83	90	82																
PNeb_wgs	74	83	72	57															
PNi_wgs	3	110	15	86	77														
PRab_wgs	77	87	75	62	9	80													
PRof_wgs	2	109	14	85	76	1	79												
PTrob_wgs	83	92	82	35	55	85	59	84											
PWin_wgs	72	83	71	58	4	75	7	74	55										
PZwi_wgs	82	91	81	34	54	84	58	83	1	54									
61VB2_wgs	11	106	10	81	72	14	75	14	81	71	80								
PBae_I_wgs	73	82	71	56	3	76	8	75	53	3	52	71							
A104S_wgs	176	114	173	165	154	179	153	178	165	154	164	176	154						
PBae_II_wgs	74	83	72	58	4	75	9	74	55	4	54	72	3	155					
PBar_wgs	84	94	82	70	18	87	20	86	68	18	67	82	17	157	18				
PBN_wgs	3	110	15	86	77	0	80	1	85	75	84	14	76	179	75	87			
PHer_I_wgs	74	84	72	58	5	77	10	76	55	5	54	72	2	156	5	19	77		
PLad_wgs	13	118	23	94	85	15	85	14	92	83	91	23	84	178	85	94	15	85	
